# Scaffolding Light-Up Aptamers on DNA Nanostructures for Fluorescence Enhancement

**DOI:** 10.1021/acsbiomaterials.5c00228

**Published:** 2025-06-18

**Authors:** Luyao Shen, Donglei Yang, Daniel Fu, Pengfei Wang, Yonggang Ke

**Affiliations:** † Wallace H. Coulter Department of Biomedical Engineering, 1371 Georgia Institute of Technology and Emory University, Atlanta, Georgia 30322, United States; ‡ Institute of Molecular Medicine, Department of Laboratory Medicine, Shanghai Key Laboratory for Nucleic Acid Chemistry and Nanomedicine, Renji Hospital, School of Medicine, 12474 Shanghai Jiao Tong University, Shanghai 200127, China

**Keywords:** RNA-based fluorescent light-up aptamers, DNA nanotechnology, aptamer functionality enhancement

## Abstract

RNA-based fluorescent light-up aptamers (FLAPs) have been progressively developed as imaging probes because of their high signal-to-noise ratio. However, it remains a challenge to use these light-up aptamers due to their poor folding and stability. Leveraging DNA nanotechnology, we investigated whether a DNA origami template could improve folding and further enhance the functionality of FLAPs, namely, the corresponding fluorescence intensities. We utilized aptamer Broccoli and its cognate fluorogen DFHBI-1T as a model. When singular aptamer Broccoli was scaffolded on DNA origami, DNA brick-based nanostructures, DNA double helices, and even on structures as simple as a DNA hairpin stem, our results showed that the fluorescence intensities could be significantly enhanced. These findings show a positive correlation between the fluorogen activity of light-up aptamers and the DNA stem length, potentially mediated by the improved structural stability of the DNA stem, as determined by their simulated thermodynamic properties. Our studies provide a new method to design and enhance the fluorescence behavior of FLAPs, especially structures with a G-quadruplex-based fluorogen recognition region.

## Graphical abstract

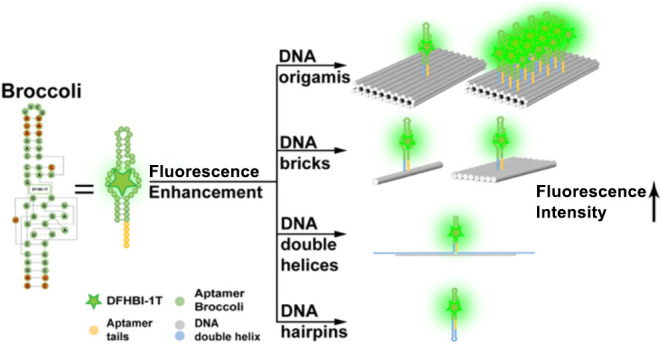

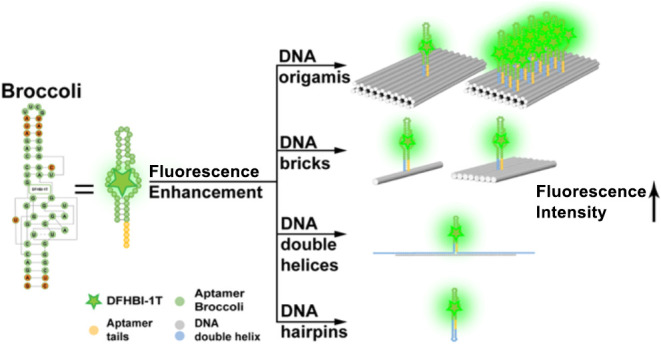

## Introduction

DNA nanotechnology has offered a variety of nanostructures with rationally designed geometric shapes and programmable features to organize molecules through precise self-assembly and addressability.^1–3^ More recently, DNA nanostructures have drawn increased interest for wide-ranging applications at the biological interface, such as structurally embedded gene expression,^4^ drug delivery,^5^ and functional enhancement of biomolecular reactions^6^ via spatially programmable and multivalent arrangements of organic molecules, including functional nucleic acids (aptamers, DNAzymes, riboswitches, etc.)^7–10^ and proteins (enzymes and antibodies).^11–15^ For example, there is a significant enhanced antibody response by the multivalent of the SARS-CoV-2 receptor-binding domain antigen displaying on an icosahedral DNA origami.^16^ Multivalent EpCAM (epithelial cell adhesion molecule) aptamers on two-dimensional rectangular DNA origami showed enhanced binding capability against EpCAM-positive cells. It also largely impaired signaling activation, resulting in cell growth inhibition.^17^ DNA nanotechnologies are expected to provide reliable and advanced strategies for designing multivalent ligands to strengthen biosensors and therapeutics.

RNA-based fluorescent light-up aptamers (FLAPs) that bind fluorogenic molecules can be used for visualizing RNA in synthetic biology applications, metabolite sensing, or monitoring dynamics inside living cells.^18–23^ They are generated via the systematic evolution of ligands by the exponential enrichment strategy (SELEX).^24^ Light-up aptamers consist of selected sequences and their cognate fluorogens. These fluorogens are cell-permeable and nonfluorescent in the free state, while, conversely, binding to the cognate aptamers restricts the free rotation to produce high fluorescence. Thus, a major advantage of light-up aptamers over other fluorescence imaging techniques (such as GFP) is the high signal-to-noise ratio. In vitro light-up aptamer-based sensors show excellent programmability. They can be precisely encoded and easily transcribed to generate needed probes.^25^ Several in vitro assays have been developed, such as in-gel imaging,^26^ miRNA detection,^27^ thermodynamic and kinetic properties of riboswitch characterization,^28^ etc. However, it remains a challenge to use these light-up aptamers due to their poor folding and stability. To improve aptamer folding, researchers have developed aptamer scaffolds, such as tRNA scaffolds,^29^ F30,^26^ and three-way junctions.^30^ Another strategy is inserting dimer or multimer aptamers into plasmids to increase the local concentration to maximize the performance as molecular tags for imaging.^31^

To leverage DNA nanotechnology, we investigated whether a DNA origami template could improve folding and further enhance the corresponding fluorescence intensities. Here, we utilized aptamer Broccoli and its cognate fluorogen DFHBI-1T^32^ as a model to characterize FLAPs scaffolded on DNA nanostructures in vitro. We combined aptamer Broccoli with a rectangular DNA origami to build single- and multi-broccoli nanostructures with different valencies and positions. Interestingly, a singular Broccoli aptamer scaffolded on DNA origami shows more than 10 times higher fluorescence than free Broccoli in the presence of DFHBI-1T, and this is the most significant instance of enhancement, even compared to multivalent Broccoli. To consolidate and investigate this fluorescence enhancement, we then connected aptamer Broccoli to different sizes of two-dimensional (2D) DNA brick-based nanostructures. The fluorescence intensity increased 10-fold even when the aptamer was scaffolded with a simple double helix template. We further reduced the complexity of the DNA nanostructure template to duplex DNA or hairpin DNA of various lengths. We found that the fluorescence enhancement happens for any doubled-stranded DNA template. Our studies offer a new route to improve the performance of light-up aptamers in addition to changing the bases or structures of aptamers themselves. It also improves the understanding about the structure and properties of other functional aptamers and offers more approaches and insights into their activity and design.

## Materials and Methods

### Assembly of DNA Origami

All of the DNA strands (Supporting Tables S1–10) were purchased from Integrated DNA Technologies. The scaffold p7560 was extracted and purified from the M13 phage variant p7560 cultured by inoculating XL1Blue in the lab. For assembly of DNA origami, 20 nM p7560 and 200 nM each staple strand (Supporting Tables 4–10) were mixed in 100 μL of 1× TE buffer (5 mM Tris, 1 mM EDTA; pH 8.0) containing 10 mM MgCl_2_. The folding mixture was annealed following this protocol: it was maintained in 85 °C for 3 min and then ramped down from 60 to 25 °C in 18 h (−0.1 °C/3 min).

### DNA Origami Purification

DNA origami was purified from excess staple strands by a 100 kDa Amicon Ultra centrifugal filter (Millipore Sigma, Burlington, MA) with 1× TE buffer containing 10 mM MgCl_2_. The sample was centrifuged 4 times at 8000 rpm for 3 min. To collect purified origami structures, the filter column was flipped into a new tube and centrifuged for 3 min at 2000 r/min. Then, the concentration was measured by the Nanodrop 2000 spectrophotometer (Thermo Fisher Scientific, Waltham, MA).

### Atomic Force Microscopy (AFM) Imaging

The morphology of DNA origami was characterized by atomic force microscopy. AFM images were obtained using a JPK NanoWizard ULTRA Speed 3 atomic force microscope (Bruker-Nano Inc., Santa Barbara, CA). 5 μL (5 nM) of samples were deposited on a freshly cleaved mica surface and left for 1 min, followed by 20 μL of ultrapure water washing. Samples were imaged under ScanAsyst mode with SCANASYST-AIR tips (resonant frequency, *f*_0_ = 45–95 kHz; spring constant, *k* = 0.4 N/m) from the silicon nitride cantilever chip (Bruker-Nano Inc., Santa Barbara, CA).

### Assembly of Double-Stranded DNA and DNA Brick-Based Structures

To prepare double helices and DNA brick-based structures (Supporting Tables 1–3), 1 μM DNA strands were mixed in 50 μL of 1× TE buffer (5 mM Tris, 1 mM EDTA; pH 8.0) containing 20 mM MgCl_2_. The folding mixture was annealed following this protocol: it was maintained in 85 °C for 3 min and then ramped down from 60 to 25 °C in 18 h (−0.1 °C/3 min).

### Agarose Gel or Native Polyacrylamide Gel Electrophoresis (PAGE)

For DNA origami and DNA brick-based nanostructures, 10 μL of DNA nanostructures mixed with 2 μL of glycerol were separated using 2% agarose gel electrophoresis at 75 V for 2–3 h in an ice water bath. Gels were prepared with 0.5× TBE buffer containing 10 mM MgCl_2_ and left to solidify at room temperature for at least 1 h before the SYBR Safe DNA gel stain (S33102, Invitrogen) was added in agarose gels for imaging under UV according to the manufacturer’s instructions. For DNA double helices, 10 μL of DNA nanostructures mixed with 2 μL of glycerol were separated using 8% native PAGE at 100 V for 1–2 h. Gels were prepared with 1× TBE buffer containing 10 mM MgCl_2_ and left to solidify at room temperature for at least 1 h. The gels were stained by the SYBR Safe DNA gel stain (S33102, Invitrogen) for 30 min after the electrophoresis was finished. Gel images were analyzed using ImageJ to quantify the band intensity of the bound complex (*I*_bound_) and any remaining unbound aptamer (*I*_free_). The binding efficiency was calculated using the equation: Efficiency = *I*_bound_/(*I*_bound_ + *I*_free_) × 100%.

### Fluorescence Measurements of Broccoli Structures

The RNA aptamers were synthesized by Sangon Biotech Co., Ltd. (Shanghai, China). The RNA aptamers were heated to 90 °C for 5 min, placed on ice for 10 min, and balanced to room temperature to form stable structures before the following steps. The corresponding concentrations of tailed Broccoli were incubated with purified DNA origami, DNA brick-based nanostructures, DNA double helices, and DNA hairpins in 40 mM HEPES (pH 7.4), 100 mM KCl, and 10 mM MgCl_2_ buffer at room temperature for 45 min. All DNA, free aptamer Broccoli, and the DNA–Broccoli complex were incubated with 10 μM DFHBI-1T in the same buffer, and then fluorescence emission was measured on a SpectraMax iD5 multimode plate reader (Molecular Devices, San Jose, CA) at an excitation of 470 nm and emission of 520 nm. The normalized fluorescence was calculated using the equation: Normalized fluorescence = *F*_complex_ – *F*_background_/F_freebroccoli_ – *F*_background._ To analyze differences in fluorescence intensities, we performed a two-tailed, unpaired Student’s *t* test for equality of means between the DNA–Broccoli complex and free Broccoli with a confidence interval of 95%. To compare the effect of DNA brick-based nanostructures, DNA double helices, and DNA hairpins on aptamer Broccoli, we ran a one-way ANOVA between the groups. To find out which groups are significantly different from each other, we then followed up with posthoc tests (Tukey’s HSD) with a confidence interval of 95%. Significance levels are denoted as follows: ****p ≤ 0.0001; ***p ≤ 0.001; **p ≤ 0.01; *p ≤ 0.05; ns (not significant) *p* > 0.05.

## Results and Discussion

### Fluorescence Enhancement of Aptamer Broccoli by DNA Origami

First, we investigated whether DNA origami could enhance the functionality of aptamer Broccoli by displaying one or multiple aptamers on the DNA origami. We designed a two-layer DNA origami rectangle (Figure 1A, Figure S1). It contains 48 positions to capture aptamers on each side, and each spot is 10 nm apart (Figure 1B). We chose 1, 4, 12, 24, and 48 spots (C1, C4, C12, C24, and C48) to connect with aptamers (Figure 1D). Based on agarose gel electrophoresis (Figure S2A) and AFM images (Figures 1C and S3), the DNA origami was successfully assembled, and the purification was completed. The sample after purification was without any leftover staple strands (Figure S2B). We then used C48 as an example to investigate how much aptamer was needed to completely occupy all of the binding sites on the DNA origami. Here, we chose the 1:1 or 1:2 concentration ratio of DNA origami binding sites to aptamer Broccoli to form an aptamer–DNA origami complex through the hybridization between the extended handles on Broccoli and those on the DNA origami. The agarose gel shows that the band of DNA origami moved slower after being incubated with aptamer Broccoli. It indicates that the connection was successful. Meanwhile, the band of two different ratios has the same distance shifts, which means that the 1:1 ratio of aptamer Broccoli was sufficient to connect with all of the spots (Figure S2C). Hence, we chose the 1:1 ratio of aptamer Broccoli to DNA origami binding sites for future experiments. Then, we incubated 100 nM Broccoli with the corresponding concentrations of DNA origami, which are 100 nM C1, 25 nM C4, 8 nM C12, 4 nM C24, and 2 nM C48. As we added more aptamers onto origami, the overall fluorescence intensities generally increased (Figure S4A). However, as a single exception to this trend, C1 exhibited the highest fluorescence enhancement, which was 10 times higher than that of free Broccoli (B) or colloidally mixed DNA origami with aptamer Broccoli (Nohandle) (Figure 1E). The multivalent Broccoli (C4, C12, C24, and C48) showed only 2 to 3 folds of per-aptamer enhancement. We suggest that this could be caused by steric hindrance when placing aptamers close to each other. To further confirm this, we designed two different patterns of captured aptamers. As Figure 1F shows, 12 aptamers would be positioned either in adjacent spots (C12_adj_) or interval spots (C12_int_). The results showed that interval placement had higher enhancement than adjacent placement (Figures 1G and S4B). It indicates that hindrances play more prominent roles than multivalent synergetic effects, which means spatial arrangement matters in a multivalent design when using aptamer Broccoli. However, based on the above results, singular aptamers scaffolded on a DNA origami template still showed the best enhancement effects. This anomaly inspired us to further investigate why the functionality of singular aptamer Broccoli could be impacted the most by a DNA origami template.

**Figure 1:**
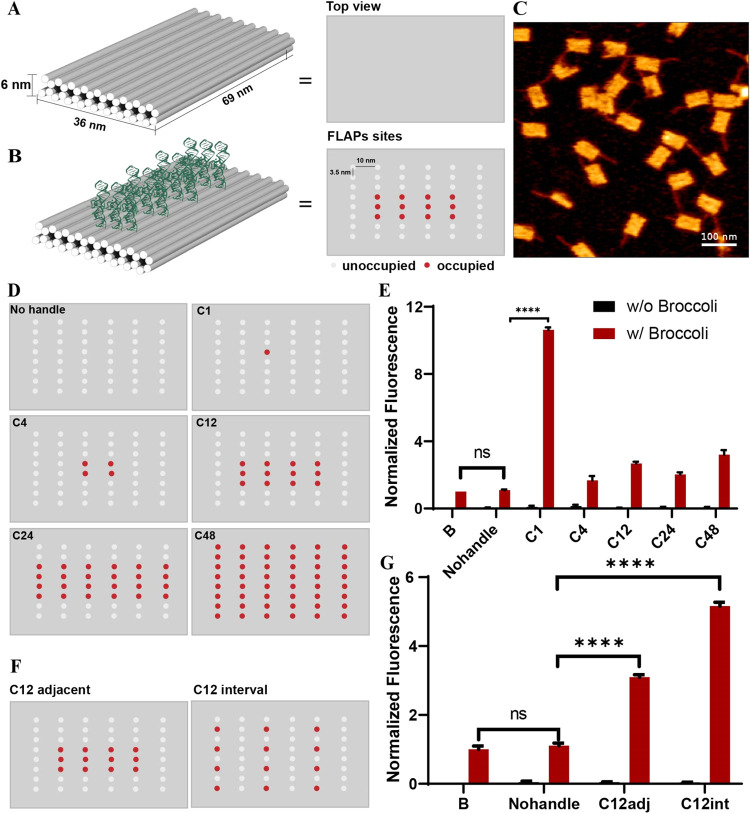
Characterization of aptamer Broccoli fluorescence enhancement by DNA origami. (A) Illustration of two-layer DNA origami rectangles and their top view. The handle length is 15nt. The interhandle distances are ∼10 nm horizontally and ∼3.5 nm vertically. (B) Illustration of aptamer Broccoli scaffolded on two-layer DNA origami rectangles. C12 is an example of this here. Red dots represent aptamer Broccoli binding sites. Gray dots represent empty spots. (C) Representative AFM images of two-layer DNA origami rectangles. The thin line connecting to DNA origami is the unused scaffold. Scale bar = 100 nm. (D) Illustration of aptamer Broccoli scaffolded on two-layer DNA origami rectangles in C1, C4, C12, C24, and C48. (E) Normalized fluorescence intensities of aptamer Broccoli on DNA origami or free aptamer Broccoli in the presence of DFHBI-1T (10 μM). The corresponding concentrations are 100 nM C1, 25 nM C4, 8 nM C12, 4 nM C24, and 2 nM C48 with 100 nM Broccoli. Free Broccoli is 100 nM. Fluorescence intensities are normalized to the same total concentration of aptamer Broccoli (100 nM), highlighting the per-aptamer enhancement effect (*N* = 3). (F) Illustration of aptamer Broccoli on two-layer DNA origami rectangles in two different patterns C12adjacent and C12interval. (G) Normalized fluorescence intensities of aptamer Broccoli on DNA origami or free aptamer Broccoli in the presence of DFHBI-1T (10 μM). The corresponding concentrations are 8 nM C12adj and 8 nM C12int with 100 nM Broccoli. Free Broccoli (B) is 100 nM. Fluorescence intensities are normalized to the same total concentration of aptamer Broccoli (100 nM), highlighting the per-aptamer enhancement effect (*N* = 3).

### Fluorescence Enhancement of Aptamer Broccoli by DNA Brick Nanostructures

We utilized simpler DNA brick-based rectangular structures to study whether the sizes of the DNA nanostructures will influence the fluorescence behavior of the aptamer. We designed four sizes of DNA rectangles (1–1, 2–1, 4–1, and 8–1) with sizes of 2nm × 22 nm, 4 nm × 22 nm, 8 nm × 22 nm, and 16 nm × 22 nm, respectively, with one aptamer located in the center of each DNA nanostructure (Figure 2A). The agarose gel shows that aptamers are fully connected with DNA brick-based nanostructures when using a 1:1 ratio of DNA brick-based nanostructures to aptamer Broccoli, and the efficiency is above 99% (Figures 2B,and S5). The graph of fluorescence intensities showed that bigger structures yielded a greater increase in the fluorescence intensities, which were 10, 12, 11, and 16 times higher than free Broccoli, respectively. In contrast, if aptamer Broccoli was only colloidally mixed with DNA nanostructures (1–0, 2–0, 4–0, and 8–0), it did not show any enhancement compared with free aptamers (B) (Figure 2C). A one-way ANOVA showed a statistically significant difference between four groups, F (3, 8) = 9.168, *p* = 0.0057. The result shows that there is strong evidence that at least one group differs from the others. Posthoc analysis using Tukey’s HSD revealed that 8–1 vs 1–1, 2–1, and 4–1 showed significant differences (*p* < 0.05); all other comparisons were not significant. These results suggest that DNA nanostructures may promote aptamer Broccoli to combine with DFHBI-1T. Additionally, the simplest 1–1 pairing already demonstrated a 10-fold fluorescence intensity increase. It indicates that only simple DNA double helices may be sufficient for enhancement.

**Figure 2:**
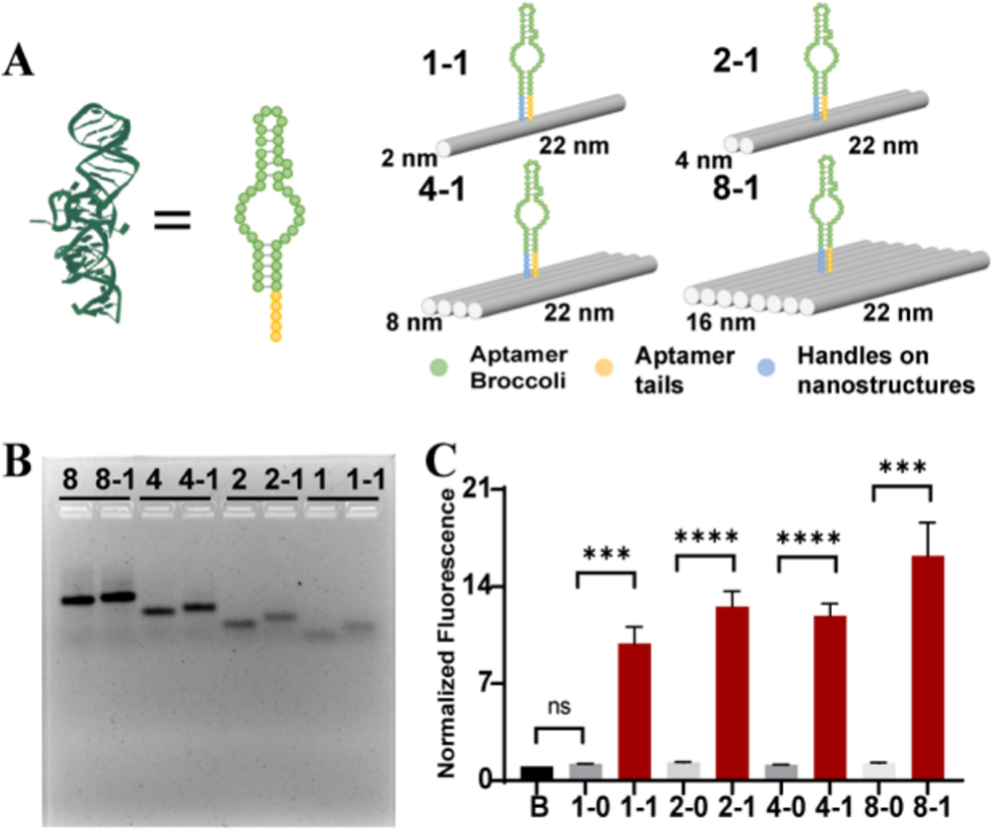
Characterization of aptamer Broccoli fluorescence enhancement by DNA brick nanostructures. (A) Illustration of aptamer Broccoli on DNA brick-based structures with different sizes. The sizes are presented as width by length. (B) Agarose gel image of DNA brick-based structures connecting with aptamer Broccoli (8–1, 4–1, 2–1, and 1–1) and without aptamer Broccoli (8, 4, 2, and 1). The band shifts indicate a combination of aptamers with nanostructures. (C) Normalized fluorescence intensities of aptamer Broccoli on DNA brick-based structures or free aptamer Broccoli in the presence of DFHBI-1T (10 μM). The corresponding concentrations are 100 nM 8–1, 4–1, 2–1, and 1–1 with 100 nM Broccoli. Free Broccoli (B) is 100 nM (*N* = 3).

### Fluorescence Enhancement of Aptamer Broccoli by DNA Double Helices

We then reduced the complexity of the DNA nanostructure templates to only DNA double helices. Singular aptamers are positioned in the middle of a single double helix with increasing lengths (M0, M3, M7, and M15) (Figure 3A). We similarly investigated the binding efficiency of different ratios of double helices to aptamer Broccoli. Through native PAGE, we find that a 1:1 ratio of the double helix to Broccoli sufficiently saturates the template (Figure S6). After incubating aptamer Broccoli with different lengths of DNA helices, the intensities displayed an increasing trend with respect to increasing lengths, showing 7-, 12-, 13-, and 14-fold enhancements of intensities, respectively. When above M7, intensities reached a plateau (Figure 3C), suggesting that a short length of duplex DNA is sufficient to stabilize aptamer folding and that further increases in the scaffold length or complexity do not result in a significantly higher enhancement. Interestingly, M0 displayed a 7-fold increase of activity between aptamer Broccoli and DFHBI-1T, yet it was only a single-stranded DNA hybridized by the 15 bp handle extension. A one-way ANOVA showed a statistically significant difference between four groups (M0, M3, M7, and M15), F (3, 8) = 4.765, *p* = 0.0344. Posthoc comparisons using Tukey’s HSD test revealed that M0 was significantly different from M3 (*p* = 0.024), M7 (*p* = 0.002), and M15 (*p* = 0.001). There were no significant differences between M3, M7, and M15 (*p* > 0.05 for all pairwise comparisons). These results suggest that M0 had a significantly different effect compared to all other dsDNA scaffolds, while M3, M7, and M15 did not differ significantly from one another. It suggested that a double helix DNA may not actually be necessary to achieve fluorescence enhancement.

**Figure 3:**
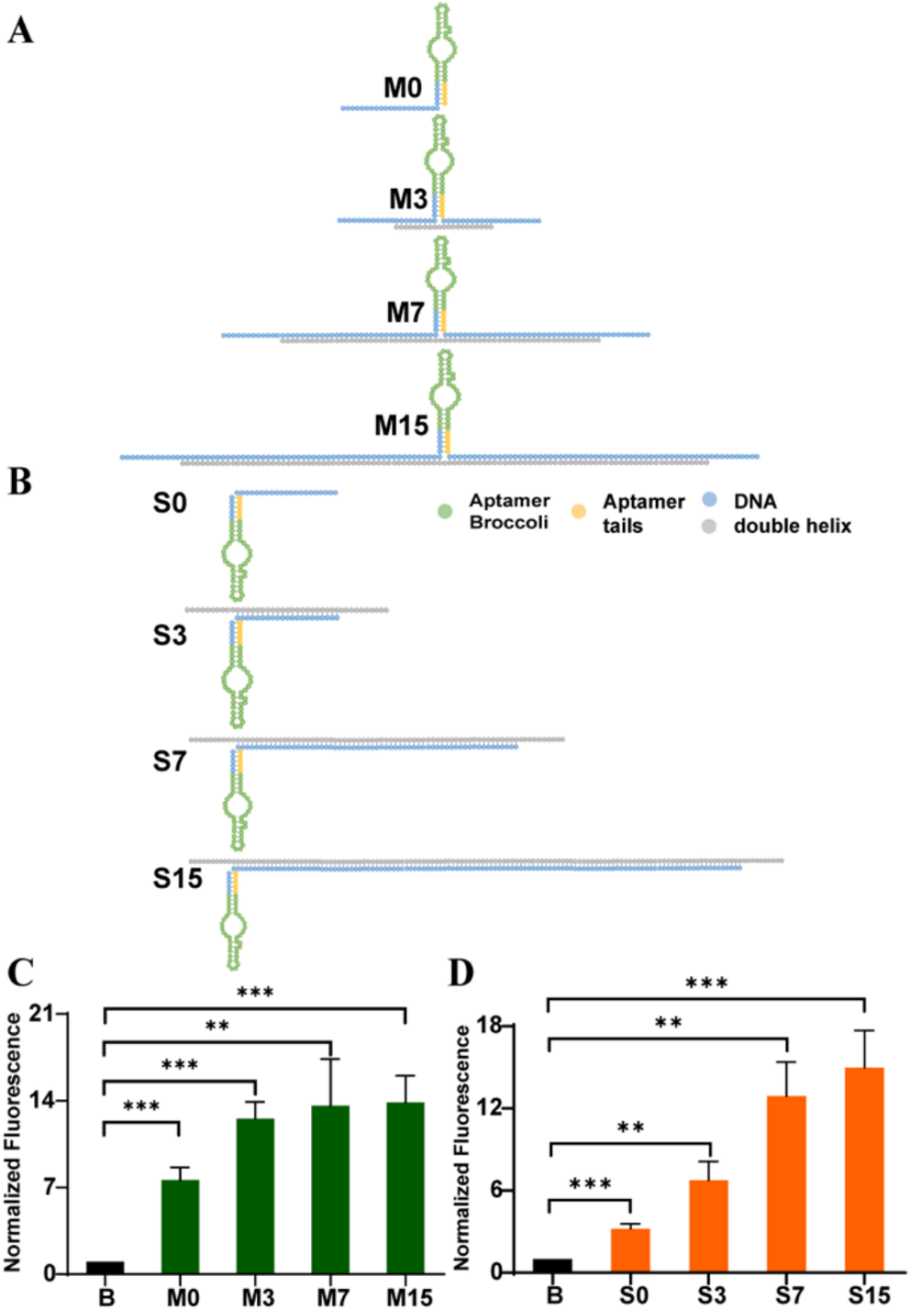
Characterization of aptamer Broccoli fluorescence enhancement by DNA double helices. (A) Illustration of aptamer Broccoli in the middle of double helices with different lengths (M0 is 0 bp, M3 is 22 bp, M7 is 66 bp, and M15 is 154 bp). (B) Illustration of aptamer Broccoli on the end of double helices with different lengths (S0 is 0 bp, S3 is 22 bp, S7 is 66 bp, and S15 is 154 bp). (C) Normalized fluorescence intensities of aptamer Broccoli on DNA double helices or free aptamer Broccoli in the presence of DFHBI-1T (10 μM). The corresponding concentrations are 100 nM M0, M3, M7, and M15 with 100 nM Broccoli. Free Broccoli (B) is 100 nM (*N* = 3). (D) Normalized fluorescence intensities of aptamer Broccoli on DNA double helices or free aptamer Broccoli in the presence of DFHBI-1T (10 μM). The corresponding concentrations are 100 nM S0, S3, S7, and S15 with 100 nM Broccoli. Free Broccoli (B) is 100 nM (*N* = 3).

These results inspired us to scaffold aptamers at the end of a double helix to assemble a long stem structure (Figure 3B). The gel shows a complete connection between aptamers and the double helix (Figure S7). After incubating aptamer Broccoli with different lengths of the stem, a similar trend was observed in which fluorescence intensities increased along with increasing duplex lengths: 3, 7, 13, and 15 times, respectively (Figure 3D). And there is a significant difference between S0, S3, S7, and S15, F (3, 8) = 22.96, *p* = 0.0003. Posthoc comparisons using Tukey’s HSD test revealed several statistically significant differences between S groups: S0 differed significantly from S3 (*p* = 0.025), S7 (*p* = 0.001), and S15 (*p* = 0.001). S3 also differed significantly from S7 (*p* = 0.001) and S15 (*p* = 0.001). No significant difference was found between S7 and S15 (*p* = 0.291). These results indicate that S0 and S3 had significantly different effects from the others, while S7 and S15 were statistically similar. Similar with previous experiments, the enhancement effect here reached the plateau statistically. Although S0 did not perform as well as M0, it still produced obvious enhancement. The above results suggest that DNA templates of any shape or scale could increase the fluorescence functionality of aptamer Broccoli. Based on the secondary structure of Broccoli, it consists of a G-quadruplex-based DFHBI-1T recognition region between two stems (Figure 5A).^33^ The duplex stem extends the terminal stem of Broccoli, which potentially will stabilize the G-quadruplex, leading to a more stable fluorogen combination. Therefore, we considered designing the DNA segment that connects with aptamers even simpler, which led to characterizing the fluorescence enhancement of the aptamer when appended to only single-stranded DNA with a secondary stem-loop structure.

### Fluorescence Enhancement of Aptamer Broccoli by DNA Hairpins

To further understand if the fluorescence enhancement of aptamer Broccoli is caused by stems stabilizing aptamer formation, we designed four hairpins with 5 (H5), 7 (H7), 10 (H10), and 15 (H15) bp-long stems, respectively (Figure 4A). For aptamer Broccoli incubated with hairpins with increasing stem length, the fluorescence intensities showed the same tendency of longer stems, leading to higher intensities (Figure 4B). H5 showed an 8-fold enhancement, while H15 was as high as a 13-fold increase. These are consistent with prior results from S0 to S15. Notably, these DNA hairpins achieve similar fluorescence enhancement while using much simpler and lower-mass DNA structures compared to larger DNA origami or brick-based nanostructures. It supports our hypothesis that the fluorescence enhancement of aptamer Broccoli is not caused by the size or the shape of the DNA nanostructure. Instead, it is due to the DNA that elongates the stem part of Broccoli to promote or stabilize the folding of the aptamer and the binding of the fluorogen to induce a fluorescence intensity increase. To confirm this, we used IDT’s OligoAnalyzer to estimate the enthalpy change (Δ*H*), entropy change (Δ*S*), free energy change (Δ*G*), and melting temperature (Tm) of the Broccoli aptamer alone and when hybridized with different DNA hairpins, duplex, and nanostructure handles. These predictions showed that aptamer–DNA hybrids with extended stem regions generally exhibit more negative Δ*G*, Δ*H*, and Δ*S* values, and the Tm changed from ∼50 to 60–70 °C compared to the free aptamer, suggesting greater folding stability (Table S11).

**Figure 4:**
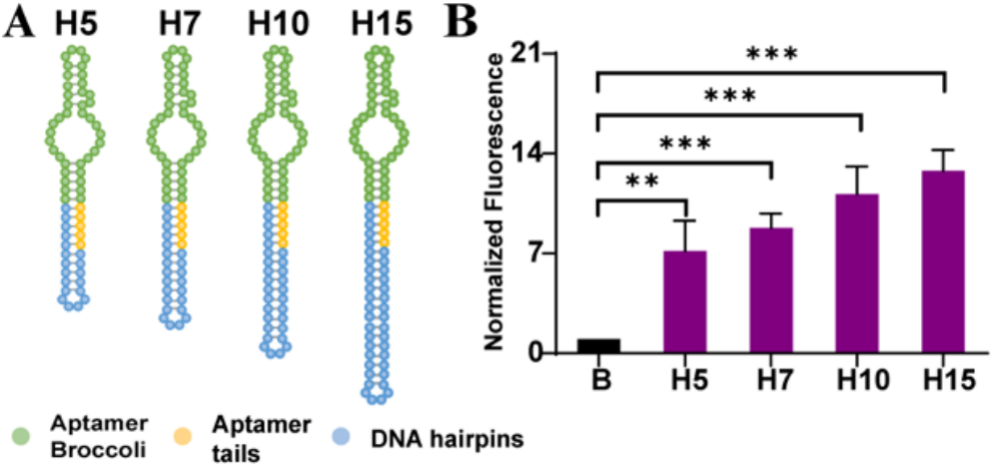
Characterization of aptamer Broccoli fluorescence enhancement by DNA hairpins. (A) Illustration of aptamer Broccoli connected with different stem lengths of hairpins. H5 has a 5-bp-long stem. H7 has a 7-bp-long stem. H10 has a 10-bp-long stem. H15 has a 15-bp-long stem. (B) Normalized fluorescence of aptamer Broccoli connecting with different hairpins or free aptamer Broccoli in the presence of DFHBI-1T (10 μM). The corresponding concentrations are 100 nM H5, H7, H10, and H15 with 100 nM Broccoli. Free Broccoli (B) is 100 nM (*N* = 3).

### Fluorescence Enhancement of Other Light-Up Aptamers by DNA Nanostructures

To demonstrate the generality of DNA templated-based enhancement effects, another light-up RNA aptamer, Baby Spinach, was used. It has a similar secondary structure and target dye-binding regions to aptamer Broccoli (Figure 5A). The same protocols for incubation and fluorescence measurements were conducted. As shown in Figure 5B, the trend of fluorescence enhancement by DNA hairpins, double helices, and DNA brick-based nanostructures is the same, which shows a 2- to 3-fold increase in intensity. However, the extent of enhancement was lower than that of aptamer Broccoli. Meanwhile, the differences among each subgroup are not significant. For example, H5 promotes enhancement similar to that of H15, as does M0 in comparison to M15. Simple DNA hairpins (H5–H15) also perform similarly as DNA brick-based nanostructures (1–1 to 8–1). While both Broccoli and Baby Spinach have G-quadruplex cores, their folding efficiency is different. Baby Spinach showed the weakest folding efficiency in vitro. And the fluorescence signals were surprisingly low using the snap cool protocol, which we used in our experiments for aptamer Broccoli.^34^ Baby Spinach might fold poorly but still has a relatively high DFHBI-1T binding affinity when it does fold. So even if DNA helps it fold a bit better, there is less gain in output signals. However, this still suggests that the structural details are not the determining factor of how the DNA template affects light-up aptamers. It also supports the aptamer stabilization hypothesis that any DNA extending the stem part of aptamer Baby Spinach will help stabilize the combination of its fluorogen and lead to fluorescence enhancement.

**Figure 5:**
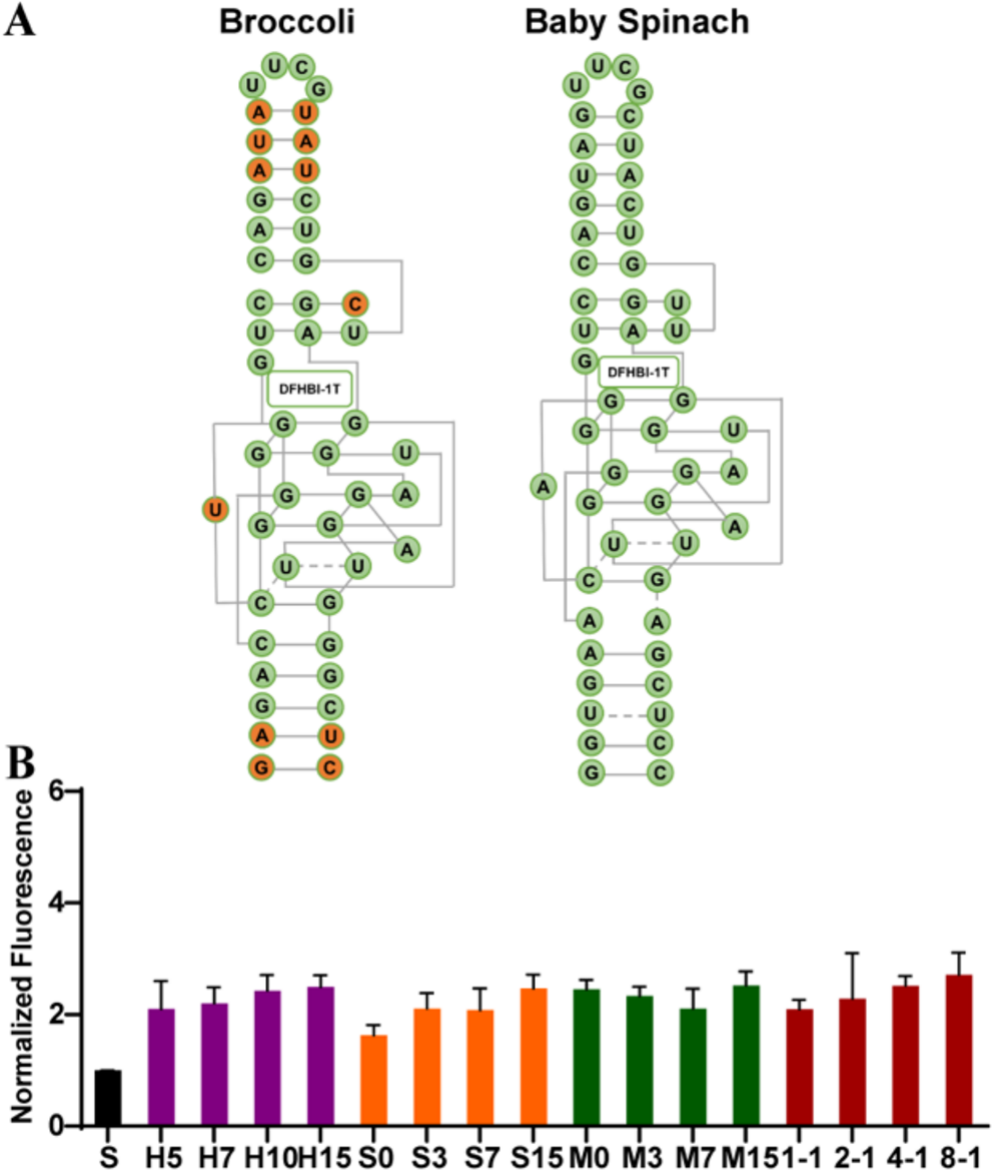
Characterization of aptamer Baby Spinach fluorescence enhancement by DNA nanostructures. (A) Secondary structures of aptamers Broccoli and Baby Spinach and the cognate fluorogen DFHBI-1T. The green bases are the same in both aptamers. The orange bases are the different bases in both aptamers. (B) Normalized fluorescence of hairpins, double helices, and DNA brick-based nanostructures (100 nM) with aptamer Baby Spinach (100 nM). Free aptamer Baby Spinach (S) is 100 nM.

## Conclusions

In conclusion, we observed the phenomenon of the fluorescence enhancement of FLAPs by DNA nanostructures. The fluorescence intensities of aptamer Broccoli with its fluorogen DFHBI-1T were significantly increased by DNA origami. However, there were more hindrance effects than synergetic effects in the multivalent arrangement of FLAPs on DNA origami. Surprisingly, the singular aptamer on DNA origami showed the most enhancement. When a singular aptamer was scaffolded on DNA brick-based nanostructures and DNA double helices, it showed a significant enhancement of fluorescence intensities as well. The results further showed that the fluorescence intensities increased when scaffolded on nanostructures as simple as a DNA hairpin stem. Other light-up aptamers, such as Baby Spinach, were affected by DNA hairpins, double helices, and DNA brick-based nanostructures as well. In the following, we hypothesize upon the biophysical mechanism by which the enhancement occurs and suggest directions for future study. The phenomenon behind it is likely due to the stabilization of the aptamer’s secondary structures. DNA nanostructures, including duplexes, hairpins, and bricks, can act as physical support that extends and stabilizes the stem regions of aptamers such as Broccoli and Baby Spinach. This reduces the conformational entropy, promoting correct folding of the aptamer. The well-folded aptamer then reduces the off rate of the fluorogen, allowing for an increased and higher overall fluorescence signal. In addition, DNA scaffolds may function analogously to molecular chaperones, guiding RNA aptamers into their functional conformations. By tethering specific points of the aptamer sequence, the scaffold can bias folding trajectories and reduce kinetic traps or misfolded states. On the other hand, when aptamers are free in solution, they may self-associate or interact, particularly at higher concentrations. Anchoring the aptamer to a DNA scaffold spatially isolates individual molecules, reducing intermolecular interference, aggregation, and nonproductive folding pathways. To further support our hypothesis, we performed simulations of the aptamers’ secondary structures and their thermodynamic parameters. The results showed that elongated stem sequences led to more negative Δ*G*, Δ*H*, and Δ*S* values and increased Tm values, suggesting greater thermodynamic stability. Similar findings have been reported in other aptamer systems, where duplex formation or stem elongation enhanced the folding efficiency and target binding.^35–37^ As shown in our study, duplex DNA adjacent to G-quadruplex-containing FLAPs such as Broccoli can significantly enhance the fluorescence, likely by promoting proper folding and structural stability. This is consistent with these findings. For instance, Krauss et al. demonstrated that stem-loop elements could improve the binding efficiency of aptamer-functionalized probes.^35^ Likewise, Miranda et al. showed that incorporating duplex arms adjacent to G-quadruplex aptamers increased their stability and fluorescence response.^37^ However, while there may be a clear relationship between the improved fluorogen activity and the increased structural stability of the DNA stem per its equilibrium thermodynamic properties, the influence of finer secondary structure motifs cannot be dismissed and should be the subject of further study. Our studies provide a new method to design and enhance the fluorescence behavior of FLAPs, especially structures with the G-quadruplex-based fluorogen recognition region. By this method, it simplifies strategies to improve the fluorescence activity of light-up aptamers by a DNA carrier instead of by mutating the aptamers themselves. It will potentially work as a codelivery system to achieve sensing or imaging in vitro and in vivo, in which targeting probes (e.g., aptamers or antibodies) and reporting probes (FLAPs) can be delivered by DNA nanostructures into cells and tissues.

## Supplementary Material



## Notes

Design diagram (CadNano) for two-layer rectangular origami structure designs based on the p7560 scaffold; agarose gel electrophoresis of DNA origami and multivalent Broccoli on DNA origami; AFM images of DNA two-layer rectangular DNA origami; native PAGE of the DNA duplex connecting with aptamer Broccoli; and sequences of all related DNA (PDF)
