# Supplementary material for: Scaffolding Light-Up Aptamers on DNA Nanostructures for Fluorescence Enhancement
Source: ACS Biomater Sci Eng. 2025 Jun 18;11(7):4068–75. doi: 10.1021/acsbiomaterials.5c00228 (PMC12264856; doi:10.1021/acsbiomaterials.5c00228)
Supplement: Supplementary file 1 [file ab5c00228_si_001.pdf]

Supplemental Information

Scaffolding light-up aptamers on DNA nanostructures for fluorescence enhancement

Luyao Shen[1], Donglei Yang[2], Daniel Fu[1], Pengfei Wang[2] and Yonggang Ke[1]**

[1]Wallace H. Coulter Department of Biomedical Engineering, Georgia Institute of Technology and Emory University, Atlanta, GA 30322, USA

[2]Institute of Molecular Medicine, Department of Laboratory Medicine, Shanghai Key Laboratory for Nucleic Acid Chemistry and Nanomedicine, State Key Laboratory of Oncogene and Related Genes, Renji Hospital, School of Medicine, Shanghai Jiao Tong University, Shanghai200127, China

Email: luyao.shen@emory.edu; yonggang.ke@emory.edu

Table of contents

Supplemental figures

Figure S1. Strand diagram (CadNano) for two-layer rectangular origami structure designs based on the p7560 scaffold.

Figure S2. Agarose gel electrophoresis of DNA origami and multivalent Broccoli on DNA origami.

Figure S3. AFM images of DNA two-layer rectangular DNA origami.

Figure S4. The fluorescence intensities ratio of aptamer Broccoli on DNA origami (F_{ori}) to free aptamer Broccoli (F_{free}).

Figure S5. 2.5% agarose gel of DNA bricks connecting with aptamer Broccoli.

Figure S6. 8% Native-PAGE gel of M7 connecting with aptamer Broccoli.

Figure S7. 8% Native-PAGE gel of S0 and S3 connecting with aptamer Broccoli.

Supplemental tables

Table S1. Sequences of DNA hairpins

Table S2. Sequences of DNA double helices

Table S3. Sequences of DNA brick-based rectangle

Table S4. Sequences of two-layer rectangular DNA origami

Table S5. Sequences of two-layer rectangular DNA origami C48

Table S6. Sequences of two-layer rectangular DNA origami C24

Table S7. Sequences of two-layer rectangular DNA origami C12 and C12adj

Table S8. Sequences of two-layer rectangular DNA origami C12int

Table S9. Sequences of two-layer rectangular DNA origami C4

Table S10. Sequences of two-layer rectangular DNA origami C1, tailed-Broccoli and tailed-baby spinach

Table S11. The thermodynamic parameters (ΔH, ΔS, ΔG and Tm) of free Broccoli aptamers and Broccoli with hairpins, duplex and nanostructure handles.

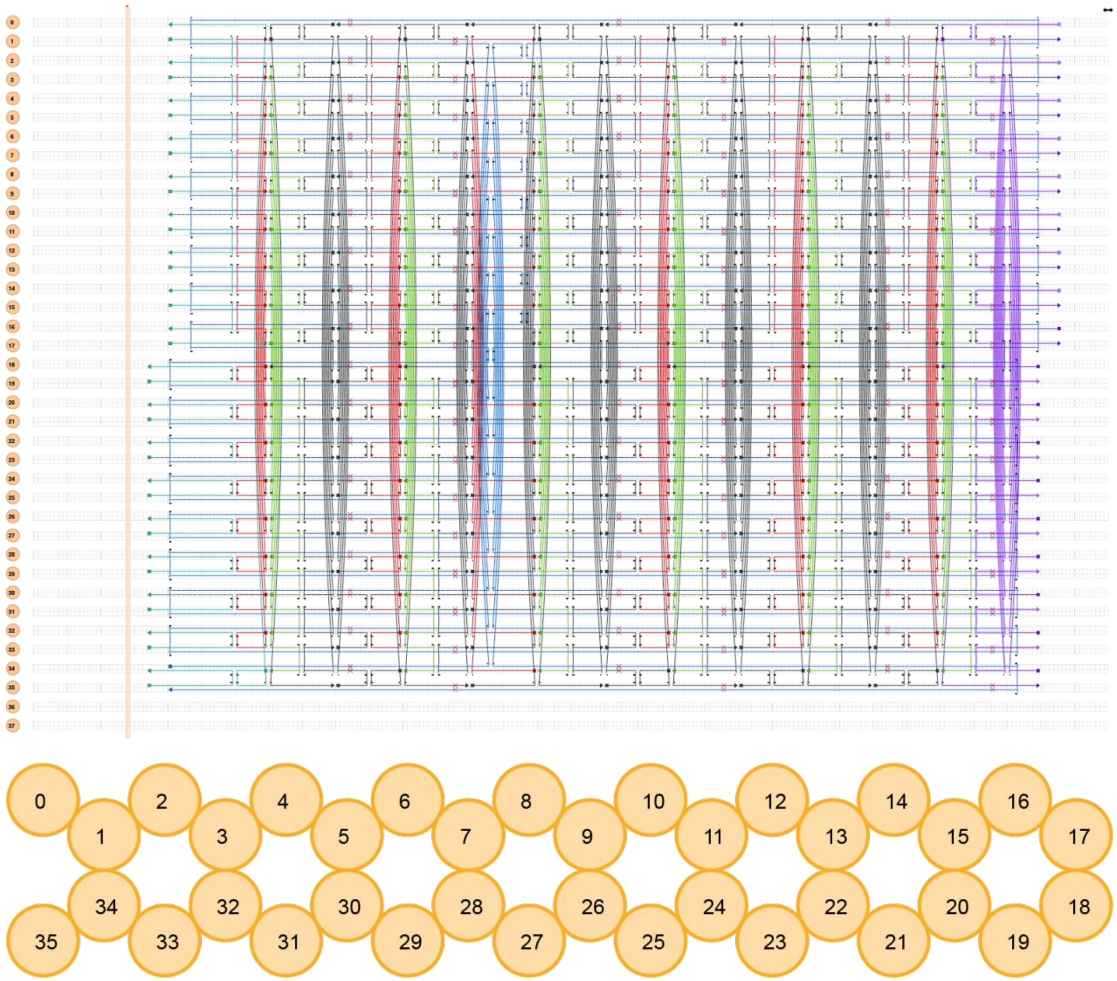

Figure S1. Strand diagram (CadNano) for two-layer rectangular origami structure designs based on the p7560 scaffold.

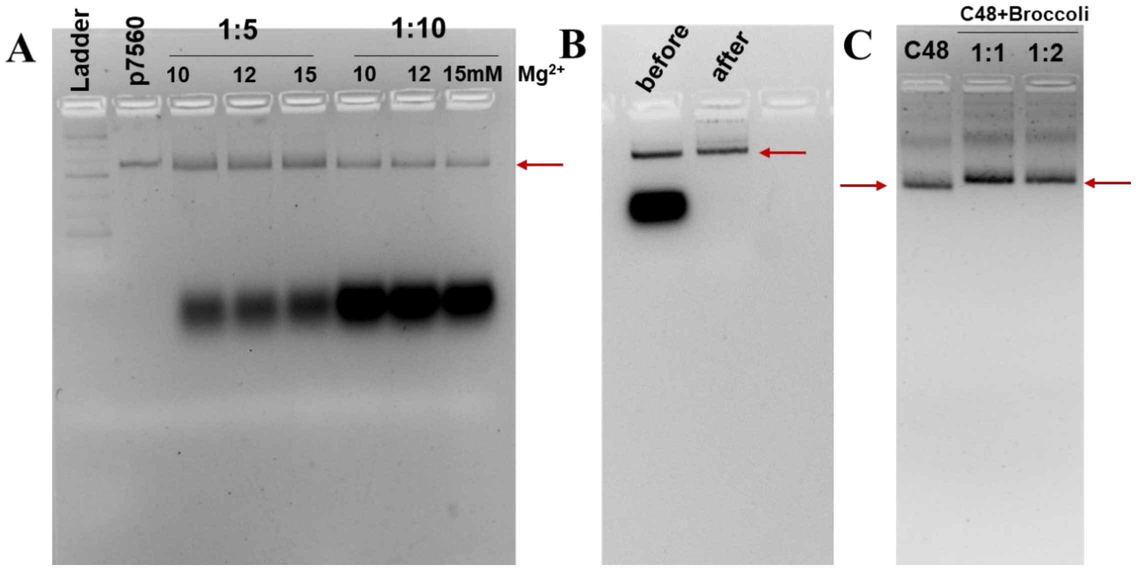

Figure S2. Agarose gel electrophoresis of DNA origami and multivalent Broccoli on DNA origami. A) 2.5 % agarose gel image of different conditions when preparing DNA origami including different concentrations of staples (10nM p7560 and 50nM staples (1:5) or 100nM staples (1:10)) and magnesium ion (10, 12 and 15mM). For future experiments, we chose to use 10 mM Mg^{2+}. B) 2 % agarose gel image of before and after purifying DNA origami by ultra-centrifuge filters (MWCO 100 kDa). C) An example gel of DNA origami before and after combining with aptamer Broccoli. The concentrations of C48 is 10nM. The corresponding aptamer Broccoli are 480nM (1:1) and 960nM (1:2). The red arrow pointed at the DNA origami.

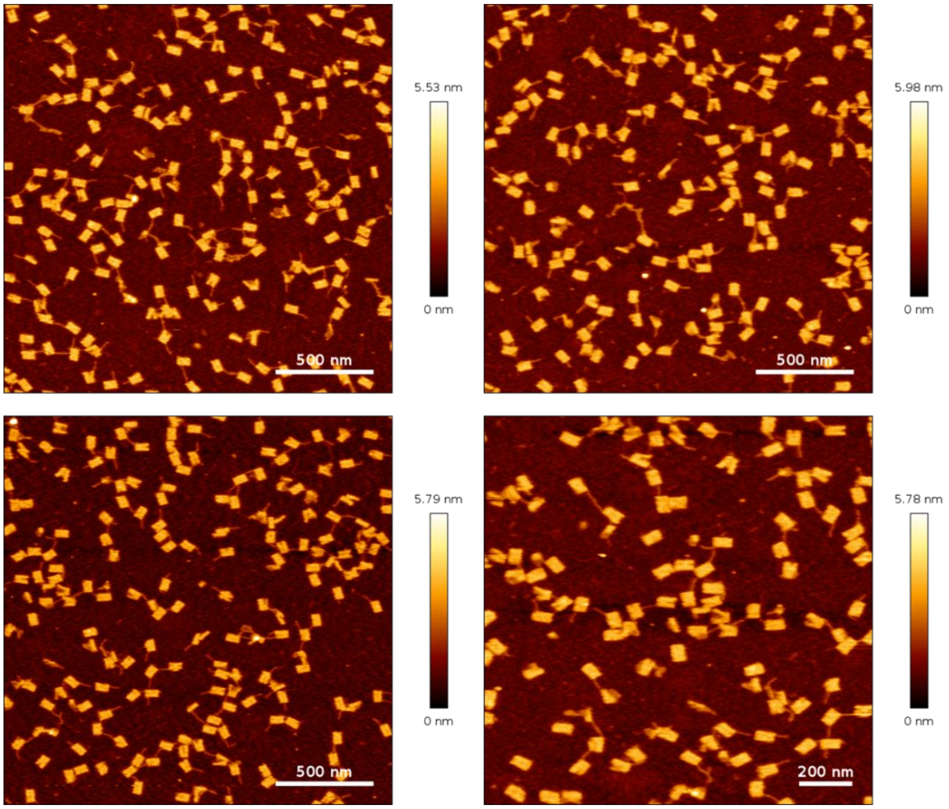

Figure S3. AFM images of DNA two-layer rectangular DNA origami.

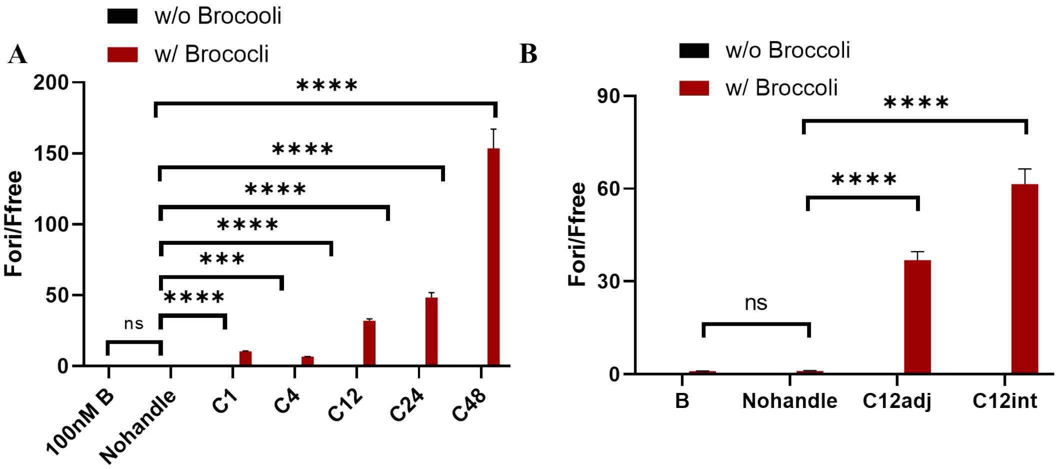

Figure S4. The fluorescence intensities ratio of aptamer Broccoli on DNA origami (F_{ori}) to free aptamer Broccoli (F_{free}). A) Aptamer Broccoli on DNA origami are positioned as C1, C4, C12, C24, and C48. The "nohandle" condition represents a control where aptamer Broccoli was mixed with DNA origami lacking complementary capture handles, preventing hybridization. The corresponding concentrations are 100 nM of C1, 25 nM of C4, 8 nM of C12, 4 nM of C24 and 2 nM of C48 with Broccoli (100 nM) in the presence of DFHBI-1T (10 μM). Free Broccoli is 100nM. B) the fluorescence intensities ratio of aptamer Broccoli on DNA origami

(F_{ori}) with different pattern (C12adj and C12int) to free aptamer Broccoli (F_{free}) in the presence of DFHBI-1T (10 μM). The corresponding concentrations are 8nM of C12adj and 8nM of C12int with Broccoli (100 nM). Free Broccoli is 100nM. (N=3) Fluorescence intensities reflect total fluorescence output, which is scaled with the number of aptamers incorporated on the DNA origami.

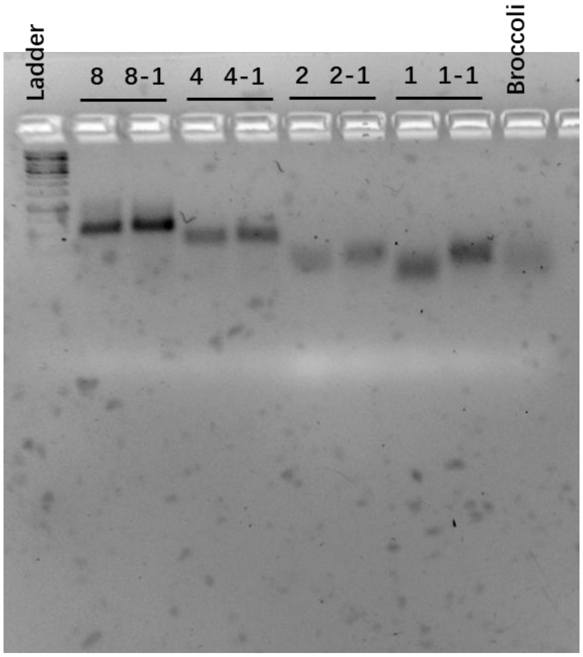

Figure S5. 2.5% agarose gel of DNA bricks connecting with aptamer Broccoli. Agarose gel image of DNA brick-based structures connecting with aptamer Broccoli (8-1, 4-1, 2-1 and 1-1) and without aptamer Broccoli (8, 4, 2 and 1).

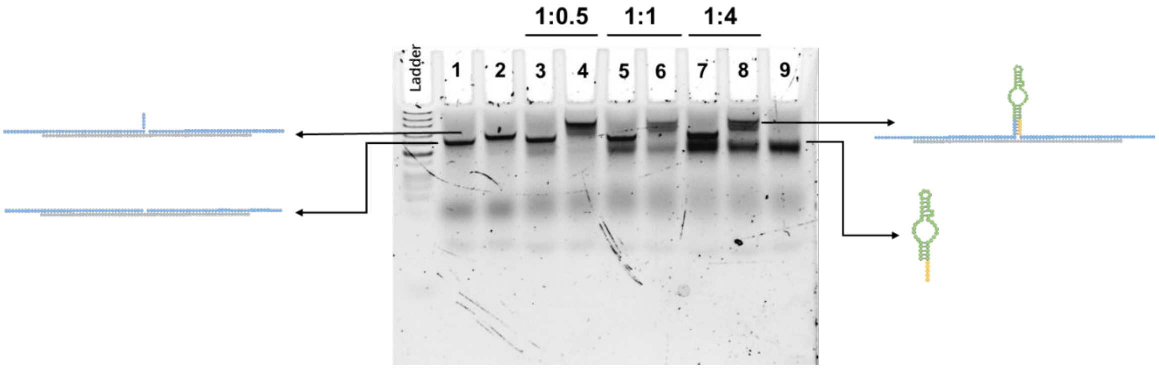

Figure S6. 8% Native-PAGE gel of M7 connecting with aptamer Broccoli. Lane 1: M7 w/o extended handles. Lane 2: M7 w/ extended handles. Lane 3: 100 nM of M7 w/o handles mixing with 50nM aptamer Broccoli. Lane 4: 100 nM of M7 w/ handles mixing with 50nM aptamer Broccoli. Lane 5: 100 nM of M7 w/o handles mixing with 100nM aptamer Broccoli. Lane 6: 100 nM of M7 w/ handles mixing with 100nM aptamer Broccoli. Lane 7: 100 nM of M7 w/o handles mixing with 400nM aptamer Broccoli. Lane 8: 100 nM of M7 w/ handles mixing

with 400nM aptamer Broccoli. Lane 9: 400nM aptamer Broccoli.

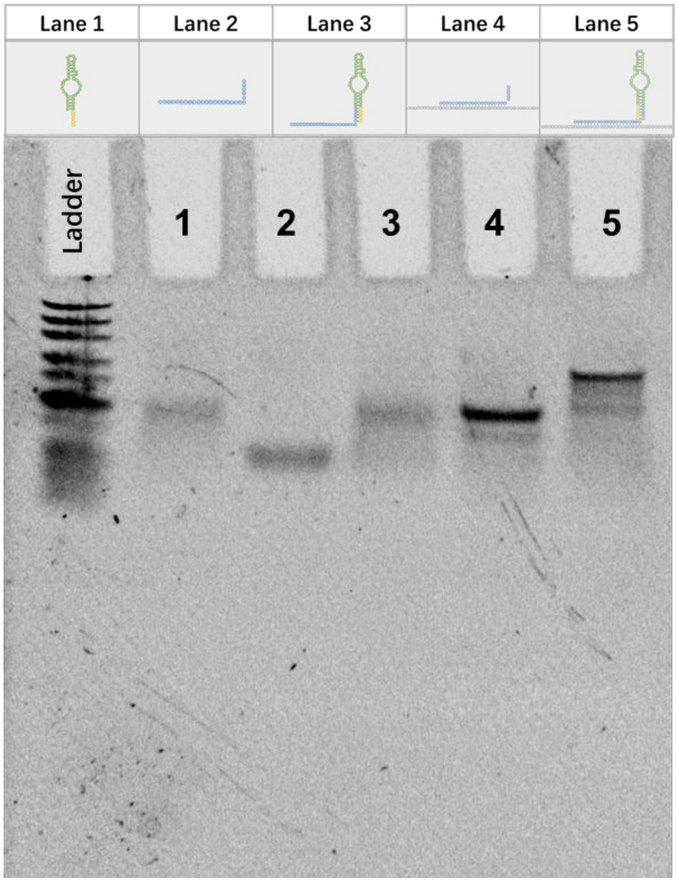

Figure S7. 8% Native-PAGE gel of S0 and S3 connecting with aptamer Broccoli. Lane 1: 100nM aptamer Broccoli. Lane 2: 100 nM S0 without aptamer Broccoli. Lane 3: 100 nM of S0 with 100nM aptamer Broccoli. Lane 4: 100 nM of S3 w/ handles without aptamer Broccoli. Lane 5: 100 nM of S3 w/ handles with 100 nM aptamer Broccoli.

Supplemental Table S1. Sequences of DNA hairpins

**Name**	**Sequences**	**Length of hairpin stems**
**H15**	CGTAGCAGAGCGACTTTTTAGTCGCTCTGCTACGTTTtcctgcacacgtcca	15
**H10**	CGAGCGATCTTTTTAGATCGCTCGTTTtcctgcacacgtcca	10
**H7**	CGAGCGATTTTTCGCTCGTTTtcctgcacacgtcca	7
**H5**	CGAGCTTTTGCTCGTTTtcctgcacacgtcca	5
**H0**	TTTtcctgcacacgtcca	0

Supplemental Table S2. Sequences of DNA double helices

**Name**	**Name**	**Sequences**

**S0**	4,31 handles	AATGCGAAAGAGTAGGAATCATTTTtcctgcacacgtcca
**S3**	4,31 handles	AATGCGAAAGAGTAGGAATCATTTTtcctgcacacgtcca
	4,0	TTTTTTTTTTATGATTCCTAC
	4,21	TCTTTCGCATTTAGTCAACCTG
**S5**	4,31 handles	AATGCGAAAGAGTAGGAATCATTTTtcctgcacacgtcca
	4,0	TTTTTTTTTTATGATTCCTAC
	4,21	TCTTTCGCATTTAGTCAACCTG
	4,43	AATACTGGGGATGGGACTAATC
	4,53	TCCCCAGTATTCAGGTTGACTA
**S7**	4,31 handles	AATGCGAAAGAGTAGGAATCATTTTtcctgcacacgtcca
	4,0	TTTTTTTTTTATGATTCCTAC
	4,21	TCTTTCGCATTTAGTCAACCTG
	4,43	AATACTGGGGATGGGACTAATC
	4,53	TCCCCAGTATTCAGGTTGACTA
	4,65	GTCACAATTGGGGACTTCCGGT
	4,75	CCAATTGTGACGATTAGTCCCA
**S15**	4,31 handles	AATGCGAAAGAGTAGGAATCATTTTtcctgcacacgtcca
	4,0	TTTTTTTTTTATGATTCCTAC
	4,21	TCTTTCGCATTTAGTCAACCTG
	4,43	AATACTGGGGATGGGACTAATC
	4,65	GTCACAATTGGGGACTTCCGGT
	4,87	CAAATCATCTAGGCGTAGAGGC
	4,109	GAACACATGAGCCATATGGTAC
	4,131	CTTCGGTTATATATCGCCCGGA
	4,153	GCATTTAGGGTTTTTTTTTTT
	4,53	TCCCCAGTATTCAGGTTGACTA
	4,75	CCAATTGTGACGATTAGTCCCA
	4,97	TAGATGATTTGACCGGAAGTCC
	4,119	CTCATGTGTTCGCCTCTACGCC
	4,141	TATAACCGAAGGTACCATATGG
	4,163	ACCCTAAATGCTCCGGGCGATA
**M0**	4,65 handles	GTCACAATTGGGGACTTCCGGTTTTtcctgcacacgtcca
**M3**	4,65 handles	GTCACAATTGGGGACTTCCGGTTTTtcctgcacacgtcca

	4,87	CAAATCATCTAGGCGTAGAGGC
	4,97	TAGATGATTTGACCGGAAGTCC
M7	4,65 handles	GTCACAATTGGGGACTTCCGGTTTTtcctgcacacgtcca
	4,43	AATACTGGGGATGGGACTAATC
	4,87	CAAATCATCTAGGCGTAGAGGC
	4,109	GAACACATGAGCCATATGGTAC
	4,75	CCAATTGTGACGATTAGTCCCA
	4,97	TAGATGATTTGACCGGAAGTCC
	4,119	CTCATGTGTTCGCCTCTACGCC
M11	4,65 handles	GTCACAATTGGGGACTTCCGGTTTTtcctgcacacgtcca
	4,21	TCTTTCGCATTTAGTCAACCTG
	4,43	AATACTGGGGATGGGACTAATC
	4,87	CAAATCATCTAGGCGTAGAGGC
	4,109	GAACACATGAGCCATATGGTAC
	4,131	CTTCGGTTATATATCGCCCGGA
	4,53	TCCCCAGTATTCAGGTTGACTA
	4,75	CCAATTGTGACGATTAGTCCCA
	4,97	TAGATGATTTGACCGGAAGTCC
	4,119	CTCATGTGTTCGCCTCTACGCC
	4,141	TATAACCGAAGGTACCATATGG
M15	4,65 handles	GTCACAATTGGGGACTTCCGGTTTTtcctgcacacgtcca
	4,0	TTTTTTTTTTATGATTCCTAC
	4,21	TCTTTCGCATTTAGTCAACCTG
	4,43	AATACTGGGGATGGGACTAATC
	4,87	CAAATCATCTAGGCGTAGAGGC
	4,109	GAACACATGAGCCATATGGTAC
	4,131	CTTCGGTTATATATCGCCCGGA
	4,153	GCATTTAGGGTTTTTTTTTTT
	4,31	AATGCGAAAGAGTAGGAATCATTTT
	4,53	TCCCCAGTATTCAGGTTGACTA
	4,75	CCAATTGTGACGATTAGTCCCA
	4,97	TAGATGATTTGACCGGAAGTCC
	4,119	CTCATGTGTTCGCCTCTACGCC
	4,141	TATAACCGAAGGTACCATATGG
	4,163	ACCCTAAATGCTCCGGGCGATA

Lower cases represent the sequences which is complementary to the extended handles of aptamer Broccoli.

Supplemental Table S3. Sequences of DNA brick-based rectangle

Name	Sequences
**1,20**	AAAGTTGTCTTTTTTTTTTTTTTTTTTTTTAACGTGTCGTG
**1,42**	TGGGGGATAGCTGAGGAGGGGGTTAACCCTTGAGCCCTGCAATG
**1,64**	AGATTTGATTGAATTCATATTGATGGGGCGGACGGACTGAGGAG
**1,86**	TTTTTTTTTTTAAGATCGGAAATGTCACTGCCTTTTTTTTTTT
**3,20**	ACAGAGACCATTTTTTTTTTTTTTTTTTTTCAATGTTCCCC
**3,42**	AGATAAGAAATCTTGTGCGCCTATTTTATCTCACTGACGCGTTC
**3,64**	AGGTCTCTGGTCCACGACGGCACGCTTCCGGTCTCTGTTTTCGG
**3,86**	TTTTTTTTTTTAGGAAGAACCACCGCCGCTCTTTTTTTTTTTT
**5,20**	AGCAGAGTAGGTTTTTTTTTTTTTTTTTTTTGGGTGAACCTC
**5,64**	TACCTGGGTGATTGTCCGAATGACTAATGATGGTTTTCCGCGTG
**5,86**	TTTTTTTTTTTCAAAAAAAAGCTGCTGCAGGTATTTTTTTTTT
**7,20**	TCGATCTGCGGTTTTTTTTTTTTTTTTTTTTATACACGTATC
**7,42**	CACAAGGTGTGAGACGTGGTATCTTAAGGTACAGTGCTTCACGA
**7,64**	CTTAGTATAGAATAGACTACCTGACTCATGTACTGCACTAGAAG
**7,86**	TTTTTTTTTTTGCTTTCTCATCGAGCGGACTTGTTTTTTTTTT
**0,31**	TCAAGGGTTAACACGACACGTT
**0,53**	GTCCGCCCCATCATTGCAGGGC
**0,75**	AGGCAGTGACACTCCTCAGTCC
**2,31**	TGAGATAAAATGGGGAACATTGAAGACAACTTTCCCCCTCCTCA
**2,53**	GACCGGAAGCGGAACGCGTCAGGCTATCCCCCACAATATGAATT
**2,75**	AAGAGCGGCGGCCGAAAACAGACAATCAAATCTTTCCGATCTT
**4,31**	AACCTTGGTCTGAGGTTCACCCATGGTCTCTGTAGGCGCACAAG
**4,53**	CCATCATTAGTAGTAAGGTGCGATTTCTTATCTTGCCGTCGTGG
**4,75**	TACCTGCAGCACACGCGGAAAAACCAGAGACCTTGGTTCTTCCT
**6,31**	TGTACCTTAAGGATACGTGTATCCTACTCTGCTGAAGCACCCGC
**6,53**	GTACATGAGTCTCGTGAAGCACCCCCAGACTCACATTCGGACAA
**6,75**	CAAGTCCGCTCCTTCTAGTGCATCACCCAGGTAGCTTTTTTTTG
**8-7,10**	CCGCAGATCGAATACCACGTCT
**8-7,32**	CACACCTTGTGAGGTAGTCTAT
**8-7,54**	TCTATACTAAGGATGAGAAAGC
**4-2,31**	TGAGATAAAATGGGGAACATTG
**4-2,53**	GACCGGAAGCGGAACGCGTCAG
**4-2,75**	AAGAGCGGCGGCCGAAAACAGA
**4,2-5,10**	CCTACTCTGCTGAAGCACCCGC
**4,2-5,32**	CCCCAGACTCACATTCGGACAA
**4,2-5,54**	TCACCCAGGTAGCTTTTTTTTG
**2,1-4,31**	AACCTTGGTCTGAGGTTCACCC
**2,1-4,53**	CCATCATTAGTAGTAAGGTGCG

**2,1-4,75**	TACCTGCAGCACACGCGGAAAA
**1-4,0**	TTTTTTTTTTGGGTGAACCTC
**1-4,21**	AGACCAAGGTTCGCACCTTACT
**1-4,43**	ACTAATGATGGTTTTCCGCGTG
**1-4,65**	TGCTGCAGGTATTTTTTTTTTT
**5,42**	TGAGTCTGGGGGCGGGTGCTTCAGACCAAGGTTCGCACCTTACT
**5,42handles**	TGAGTCTGGGGGCGGGTGCTTCAGACCAAGGTTCGCACCTTACTTTTtcctgcacacgtcca
**4,21handles**	AGACCAAGGTTCGCACCTTACTTTTtcctgcacacgtcca

Lower cases represent the sequences which is complementary to the extended handles of aptamer Broccoli.

Supplemental Table S4. Sequences of two-layer rectangular DNA origami

**Name**	**Sequences**
**0[104]**	AGGATCCCCGGGTACCGCGGATTGACCGTAAT
**0[136]**	ATGAATCGATAGCTGTCAACCCGTCGGATTC
**0[168]**	CAGTCGGGCTCACAATAGCTTTCATCAACATT
**0[200]**	ATTAATTGCATAAAGTAAAATAATTCGCGTC
**0[72]**	GGCTGCGCGCTTGTTAGTGTAGATGGGCGCA
**10[104]**	ACCGCACTCATCGAGAGCCAACAGTAGGGCTT
**10[136]**	ATGTAAATATATGCGTGTTTAGAGAGGCTT
**10[168]**	ACAAAGAATCATAATTAATCGTCATTACCAGA
**10[200]**	TTTTAGTTATAAGGCAATGCTTTATAGTAA
**10[72]**	CAAAATCACGCTATTTTTTTAAACGCCAA
**12[104]**	CCAGAAGGAAACCGAGTCAATAAACAGCCATA
**12[136]**	TATTTTCAGCTAACGGGGAAGAACTTATGC
**12[168]**	AATAGCAACCAGCTACTAACGGAAGTTTAATT
**12[200]**	CCGGTATTCCTTAAATCATCAGTCCAGAAC
**12[72]**	TATCATTCCAATAGAAATTCTGTAACGATT
**14[104]**	CTCAGAACCGCCACCCACTATTCATTAAAGGT
**14[136]**	GGAATACCTTCAACCCGGTGTACTCATAAG
**14[168]**	CTCCTTATGGTTTACCACCTTCATTTACTTAG
**14[200]**	AAAATACATATTTTGGATATTCAAATTGTG
**14[72]**	AGAAAAGTAGATAACTAGACGGGGCCATTT
**16[104]**	GTAACACTGAGTTTCGTTTGCCCGTATAAACA
**16[136]**	CAGAGCCGCTGGTAACGTAATGCCTAAAGA
**16[168]**	GCCGCCAGTCATACATACGAAAGAGAGGGTAG
**16[200]**	CAGACGATGCAGTCTCATCTTTGCACCCTC
**16[72]**	CACCGGAAGCGCGTTACCATCGAAACCTAT
**17[119]**	CAAAAGGAGCCGATATGGGTTTTAATTGTAT
**17[151]**	CAACGCCTGAAAATCTTTCGAGGTGAATTTC

**17[183]**	ATAGTTAGAAAGGAATCCGATAGTTGCGCCGA
**17[215]**	CTTTCCAGTTCAGCGCGCCCACGCATAACCG
**17[87]**	GGATAGCACACCGTACTCAGTACCAGGCGGA
**18[120]**	CTTTTTCACAGCTTGCTCCAAAAAAAAGGCTC
**18[152]**	CAACGGCTCTTGATATGCGAATAATAATTTT
**18[184]**	AGCAGCGAACAACCATGAGTGAGAATAGAAAG
**18[56]**	TATTCTGAGGGTTTTGCTCAGGAGGTTTAGTA
**18[88]**	GTTAATGCGTCGAGAAAGTATAGCCCGGAAT
**19[103]**	GAATTATCAGTAGCGGTTTGCCTATGTACC
**19[135]**	GGAACCGAGTAAAATATAAGTTTTACAAACTA
**19[167]**	CCGGAACGACCTAAAGGCTTTTGAGCCCTC
**19[199]**	TCGAAATCAAAACACTCTGAATTTTTTGTCGT
**19[71]**	GGGAATTACCAATGAATTCATCGGATTTTCAG
**21[103]**	TTATTTATCCTGAACGGTAATTGCGCCACC
**21[135]**	GATTTTAACAGATGAAGATTGAGGACCACCCT
**21[167]**	TCAACTTTCTGGCTGAGCGCCAACAGAGCC
**21[199]**	GAGTAGTAAAGAACCGTCACAATCAGGCAGGT
**21[71]**	TTTTGTTTAAGCGCATCCACAAGAAGAGCCAC
**23[103]**	AATTGAGAAACAACAATGCAGAAAAAGTTA
**23[135]**	TTGCAAAAAGGACGTTAGCGTCTTATAATAAC
**23[167]**	CGACGATAAACGAACAATTTTATATTAAGA
**23[199]**	GAGCAACAAGAAAGATTCAAGATTAAACGTAG
**23[71]**	CATGTAATGGTAAAGTTAAGTCCTCCTTTTTA
**25[103]**	GAGGCGAAATCAATAAATAACCTACCAAGT
**25[135]**	ATTCGAGCTAGTAAAATTATACAAGCCGTTTT
**25[167]**	AAGCCCGAACTGCGGACTAGAAACGCGCCC
**25[199]**	GCAAAGCGCCCCCTCAGTTAAATAAGGCTTAT
**25[71]**	AAAAGAAGGAATTACCAATTAATTTCTTTCCT
**27[103]**	ATGAAAAACTAACAAAGAGCCGTCCTTTTT
**27[135]**	AGATACATGCAAACTCTAACGGATATAACTAT
**27[167]**	TCTGCGAACTTTAATGTAACAGTTCGCAAG
**27[199]**	AGTTTCATTTTGCGGAGATTTTCACAAATATA
**27[71]**	CTCAAATAGAGGAAGGTTAGAAGTGAATTTAT
**29[103]**	TAAACAGGGCAATACAGTAATAAAAAGAAA
**29[135]**	GATAAAAATTTAGCTACAGTATTATCATATTC
**29[167]**	TTTGCGGGAAAAGGTCCAGCAGAATCAATA
**29[199]**	TTGTACCAAGCATTAAAAACATCGTTCTGAAT
**29[71]**	CAGGAACGGAGTCTGTCTCAAACTAAAGTTTG
**2[104]**	CAAGTTTTTTGGGGTCTATGTGGTTTTTCTTT

**2[136]**	TCATGGTCGCCAACGGAAAAGCCTCGTAAA
**2[168]**	GTTATCCGAAACCTGTGCAAATATCTGGAGCA
**2[200]**	GCCGGAAGCGTTGCGGTTAAAATTACAAAG
**2[72]**	TACGTGGTAACTGTTAGCTTTCCGATTGCC
**31[103]**	TCACCAGTGCAAAATTCAAAAGACTGAAAT
**31[135]**	ACTAGCATTAAATGCATGACGAGCCCAGTCAC
**31[167]**	AACAAGAGGATTCAACGCCGCTACTGGCCA
**31[199]**	GCTATCAGTCAAATCAGCGCGTAAGAAAGCGT
**31[71]**	CTTCACCGGAAAATCCTTGTTCCAATGGAAAT
**33[103]**	GGGATAGGCCGGAAACGCCATTCCCCAAAT
**33[135]**	TCCGTGGGGATAATCACGCGGGGAGTAAAGCA
**33[167]**	AAATGTGATGTATAACGTGCCAGCCCCGAT
**33[199]**	TGGCCTTCAATATTTTCTCACTGCCGAACGTG
**33[71]**	TCGTAACCCTCCAGCCGGGAAGGGATCAGGGC
**35[103]**	TTCTGGTGTCACGTTGCCTCGATAAAGACGG
**35[135]**	CCCCGGTTAACAAACGGAGCTCGAATTCGTAA
**35[167]**	AGGAAGATGCGAGTAATTCCTGTGTGAAATT
**35[199]**	TAAACGTTCTGTAGCCTCCACACAACATACGA
**35[71]**	AGATCGCAGTGCATCTTAAACAGGGCTTAAGC
**4[104]**	GGATTATTTACATTGGACCTCAGAGCGGGAGC
**4[136]**	CTAAATCGGTTGCTTATGCCTGAACGCAAG
**4[168]**	TTAGAGCTGCTTAATGAAGGGTGAGTAATACT
**4[200]**	GCGAGAAATCACGCTCCATCAATAAATCGG
**4[72]**	GATGGCCCTTGAGTGTGTTTGATTTTTAGA
**6[104]**	CCACCAGAAGGAGCGGTGGGAGCCAGCAGCAA
**6[136]**	ACGACCAGAGGCGGTTATTTTCAGACCATT
**6[168]**	ACAGAGATACGAACCAGGCATCAATTCCCAAT
**6[200]**	AAGAATACAGCCCTACATCCAATGTCTGGA
**6[72]**	ACCTACATAGAAGAACCATCACGGCTGAAC
**8[104]**	AACCTCCGGCTTAGGTGAAACAAAATCGCGCA
**20[56]**	GATTAGCGAACATGAACCACCCTCCATTTTCG
**20[88]**	TAAGTGCCCCCCTGCCAGGAACCCTTAGCGTC
**20[120]**	CGGTTTATTGAGGAAGTCACCAGTAACGGGGT
**20[152]**	TTAAACAGACAGAGGCCCACAGACATGATACA
**20[184]**	CAATGACAAAGACAGCTCTAAAGTACCGTTCC
**20[216]**	ATATATTCGCTTTTGCAAATGAATAGCCAGAA
**22[56]**	AAACGTCAGAGCCAGCCCGGAACCATTGAGTT
**22[88]**	CCGTAATCACCGTCACCTCAGAGCAGCGCTAA
**22[120]**	TTAAACGGACTGACCATCAGAGCCGAGGGAAG

**22[152]**	AGGCACCAAGGCGCAGACCACCACAGACAAAA
**22[184]**	AATACACTCGCGACCTGGAGGTTGAATAGAAA
**22[216]**	CGATTATAGTATCATCATATTCACCACCACGG
**24[56]**	AAACAGGGAACGTCAAACCGAAGCGAACAAGA
**24[88]**	CTGAACACCCCAATCCAGCCGAACCGCGCCTG
**24[120]**	AAAGAGGAGAACTGGCGAAACGCATCCAGAGC
**24[152]**	CGCATAGGAATCATTGCTGGCATGCCTGAATC
**24[184]**	ATCTTGACAATTGGGCATGTTAGCAGTTGCTA
**24[216]**	ATCAACGTGCCCTGACGGTGGCAAGCGGGAGG
**26[120]**	TACCAGTCGAAGTTTTACAAGCAAATTCTTAC
**26[56]**	CGACAAAATTAGGCAGATCGGCTGTTCCCTTA
**26[88]**	CGACAATAATCGCCATGGTATTAATGCTTCTG
**26[152]**	GTTAATAAAAAACCAAATCATTACAAGCCTGT
**26[184]**	TTACAGGTCTATCATAGATATAGAAGAATAAA
**26[216]**	AGGAATACAAAGGAATGCGAGGCGACCGACCG
**28[56]**	TTTCATTTATGATGAATCAATAGTATTAGACT
**28[88]**	GTACATAATTATTCATAGAGACTACAATAGAT
**28[120]**	GGGGGTAATTCAAAGCTGGGTTATTCGCCTGA
**28[152]**	CGTCCAATAAGACTTCAAATCCAAACCTTTTA
**28[184]**	CATTGAATGATTGCATAACTTTTTGGTTTAAC
**28[216]**	CAGAAAACTACCCTGACTTCTGACAACAGAAA
**30[56]**	AAGGAATTTCAAACCCTAATTTTAATCGGCCT
**30[88]**	TAGGAGCATCTAAAGCTGCGGAACCATCACTT
**30[120]**	GACCGGAATTCGCAAAAATTATCAACACCGCC
**30[152]**	GAGAGTACCGAGTAGAGGCAATTCAGATAAAA
**30[184]**	AGGTCATTTCCATATAGATTATACCCATTAAA
**30[216]**	TGCTGAATGCAACTAACCTACCATTAATGCGC
**32[56]**	GAGTAAAAGTACGCCAAAACGCTCGTTTGGAA
**32[88]**	CCGTTGTAAGGCCGATTCAATCGTATAGCCCG
**32[120]**	ATAACCTGTTTTTAGACAGATTCAACGTATAA
**32[152]**	GCGAGCTGAGAAGCCTGGGACATTCAGGGCGC
**32[184]**	ATAGTAGTAAAACATTTCTGACCTCCACCACA
**32[216]**	CAGGCAAGCTCAGAGCGACAATATCTGGCAAG
**34[56]**	CAGCAGGCCCTGGCCCAACCGTCTCGATCGGT
**34[88]**	CGAAATCGGAGACGGGAACCATCAGCCATTCA
**34[120]**	ATATATTTGTCAATCAGAGGTGCCGAGGCGGT
**34[152]**	TAGGTAAAAATCGATGAAGGGAGCCTGCATTA
**34[184]**	GGAGACAGGTCATTGCAAAGCCGGCCGCTTTC
**34[216]**	CAACCGTTCTATTTTTAGAAAGCGTAACTCAC

**1[55]**	CAAGAGTCGGGCGAAATGAGAGAGCCTCAGGA
**1[87]**	AGATAGGGACTACGTGCAACAGCTGGCACCGC
**1[119]**	TTGCGTATTGGCAAAGCCAGGGCGCCAGGGTA
**1[151]**	GTACTATGGAACCCTAAACGGTAACCAAAAAC
**1[183]**	CCCGCCGCTGACGGGGCTGAGAGTTTAAATTG
**1[215]**	TGTAGCGGGGAAGGGAGAGAGATCTCGCATTA
**3[55]**	TGCTGGTAAACAGGAAGAATCCTGGTTTGCCC
**3[87]**	GCCTGAGTTTTGACGCTAAAGGGAGGTGGTTC
**3[119]**	CGTGCTTTCCTATAAACCCTTCGTTAGAACTC
**3[151]**	CAGAGGTGTAATAAAATTATTTCAGTAATGTG
**3[183]**	AATACCGAAGAACCCTATGACCCTGAAAGGCC
**3[215]**	GAACTGATGTGGCACAATAAAGCTATGATATT
**5[55]**	TTACAAACAACGTTATTCAATCAAAGGCCACC
**5[87]**	AATACATTTATCATTTATCACCTTCAAATTAA
**5[119]**	TGCAACAGTGCTGATTTTCTTCACGCTGATCA
**5[151]**	CATCGGGACAGATGATTTTAGTTTTTTGGGGC
**5[183]**	GTCAGATGATTGTTTGACAGTTGATTCTACTA
**5[215]**	TAAAGAAAGGTTAGAAAGTACGGTAAATCATA
**7[55]**	GAATCCTTGAGAAGAGACAAACATACAGTTGA
**7[87]**	TAAATCGTTAGGTCTGTTCAATTAAATATCTT
**7[119]**	TTGCTTTGAATAGATTCTAATACCAAGTTCCA
**7[151]**	TTAGTATCGCTGATGCAAATATCGCAGGATTA
**7[183]**	CACCGGAACGCGAGAACAAAAAGATTGATAAG
**7[215]**	TGTGATAAAATTTCATCTATTATAAGCTTAAT
**9[55]**	AAAATAATAATCAATAAGGCATTTTTTAACAA
**9[87]**	TTTATCAACAAGAACGATTTAACATGGAAACA
**9[119]**	CAGTATAAAGCGAGTGTATGTCAACGCTCAGA
**9[151]**	TTACCAACTCGTAGGAAATAGCGACTGGATAG
**9[183]**	TTTTGCACGCAAATCAACCCTCGTTAAATATT
**9[215]**	TTTTGAAGCTAAGAACTACGAGGCAAACAGTT
**11[55]**	AAGCCCAAGCTATCTTAAATGAAATAAAGTAC
**11[87]**	TATCAGAGAAGCAGATAAATAAGACCAGACGA
**11[119]**	CTAATTTGCCAAGCTATGTTCGTTACAAATTA
**11[151]**	GGGCGACACAAAAGAATGAATTACAAATCTAC
**11[183]**	ATTCATATTACGCAGTTTGAGATGCAACATTA
**11[215]**	AATAAGTTTACATAAAGAGAAACATGAGATTT
**13[55]**	GTCATAGCCAAAATCAAAAATCACTAACATAA
**13[87]**	AGACTGTACCGCCTCCCGACTTGAAGAATTAA
**13[119]**	GTAAATATTGACAGAGAAAGTCGGAAATTTTG

**13[151]**	GGAGTGTACCACCAGAACGGTCAAAGACCAGG
**13[183]**	AGTAAGCGCATTGACAGCTCCATGCAAGAGTA
**13[215]**	TGGAAAGCTGGCCTTGGCCTGATATTACCCAA
**15[55]**	CCGCCACCCAGAGCCAAGTATTAAAAGGCCGG
**15[87]**	AGGTGTATAGCCCAATTATTTCGGTAGCAGCA
**15[119]**	CAGTGCCTTGAATCAAACAGAGTAACAGTCCA
**15[151]**	TTCACGTTGTAGCATTTTTGAGGACACTACGA
**15[183]**	GAACAACTCGTAACGAATCGGAACGGCAAAAG
**15[215]**	TCAACAGTACGTTAGTGGGATCGTACCCCCAG
**0[32]**	GCGGGCCTGTAATGAGGCCAGTTTGAGGGGACGACTTTTT
**2[32]**	TTTTTGACAGTATCGGTTGCAGCAAGCTTTTT
**4[32]**	TTTTTGGTCCACGCTGAGAAGTGTTTTTTTTT
**6[32]**	TTTTTTATAATCAGTGTATCTGGTCAGTTTTT
**8[32]**	TTTTTTTGGCAAATCACAAGAAAACAATTTTT
**10[32]**	TTTTTAATTAATTACATCGAGCCAGTATTTTT
**12[32]**	TTTTTATAAGAGAATAATAGCAGCCTTTTTTT
**14[32]**	TTTTTTACAGAGAGAACAGTAGCACCATTTTT
**16[32]**	TTTTTTTACCATTAGCGAGGCTGAGACTTTTT
**17[55]**	TTTTTTCCTCAAGAGAAGGATTAG
**18[27]**	TTTTTTCAGAACCGCCACCCTCTCAGAACCGCCACCCTTTTT
**20[27]**	TTTTTGCCATCTTTTCATAATCCCCTTATTAGCGTTTTTTTT
**22[27]**	TTTTTCAATGAAATAGCAATATAATAAGAGCAAGAAATTTTT
**24[27]**	TTTTTCGAGCATGTAGAAACCATCCCATCCTAATTTATTTTT
**26[27]**	TTTTTCTTAGATTAAGACGCTGAAAACATAGCGATAGTTTTT
**28[27]**	TTTTTTTAAATCCTTTGCCCGAATTCGACAACTCGTATTTTT
**30[27]**	TTTTTTACCGCCAGCCATTGCATATCCAGAACAATATTTTTT
**32[27]**	TTTTTGGACTCCAACGTCAAACACTATTAAAGAACGTTTTTT
**34[27]**	TTTTTTGACCTCCTGGTTGGTCTTCGCTATTACGCCATTTTT
**1[244]**	TTTTTAATAGCTCATTTTTTT
**3[244]**	TTTTTGCGATAAATTATTTTT
**5[244]**	TTTTTCTAATTAGCAATTTTT
**7[244]**	TTTTTTTTTGTAGCTCTTTTT
**9[244]**	TTTTTTGGACCATAAATTTTT
**11[244]**	TTTTTCCTAACTAATGTTTTT
**13[244]**	TTTTTAAACTGCTCATTTTTT
**15[244]**	TTTTTATCCGAAACAATTTTT
**17[244]**	TTTTTGGAGAGGCTTGTTTTT
**18[216]**	TTTTTCAGGGAGTTAAAGGCCGGTCGCTTTTTGCTAAACAACTT
**19[239]**	TTTTTAGTACAACGGAGATTTCCAAGCGCTCATTAATTTCTGTATGTTTTT

**21[239]**	TTTTTTCAGTGAATAAGGCTTAACAAAGCGCAAAGAAAACAAATAATTTTT
**23[239]**	TTTTTCAGATACATAACGCCACACATTCCCCGACTTCATATAAAAGTTTTT
**25[239]**	TTTTTTCAAAAATCAGGTCTTGAGAATGTTTGAAATTTTTAGCGAATTTTT
**27[239]**	TTTTTAACATGTTTTAAATATATAATGCGCACGTAACTAAATTTAATTTTT
**29[239]**	TTTTTAATTAAGCAATAAAGCGCAAAGATTAGTCTTATCAAAATTATTTTT
**31[239]**	TTTTTATGCCGGAGAGGGTAGCTAGCTGCTAGGGCGTTTTGAATGGTTTTT
**33[239]**	TTTTTTTTTAACCAATAGGAATTAAATCGAGTGAGCAAAGGAGCGGTTTTT
**35[239]**	AATTTTTGCGCCATCAGTAAAGCCTGGGGTGCCTTTTTT
**36[104]**	TAAAACGACGGCCAGTTGCCATCTGTAAGCAA
**36[136]**	AGAAGCCAGGGTGGATGGAGTGACTCTATGAT
**36[168]**	GTGAATTCATGCGCACAACCCCGCTTCTAATC
**36[36]**	GCGAAAGGGGGATGTGCTGCCACATAAATCATTTCTCCGA
**36[72]**	GTAACGCCAGGGTTTTAATATAGGGGCCTTGA
**37[103]**	CTCGTCGGTGGGCACGCCCAGTCACGACGTTG
**37[135]**	ACCGACAGTGCGGCCCGCCAAGCTTTCTCAGG
**37[167]**	TATTTACGCTCGCCCTGTTCTTCTAAGTGGTT
**37[207]**	CTTATGACAATGTCCCGCCAAAATGACTTAAGTGTCCTTAGTGCTGAA
**37[71]**	ATCGGCTGACGCATTTAAGGCGATTAAGTTGG

Supplemental Table S5. Sequences of two-layer rectangular DNA origami C48

**Name**	**Sequences**
**1[55]**	CAAGAGTCGGGCGAAATGAGAGAGCCTCAGGAtcctgcacacgtcca
**1[87]**	AGATAGGGACTACGTGCAACAGCTGGCACCGCtcctgcacacgtcca
**1[119]**	TTGCGTATTGGCAAAGCCAGGGCGCCAGGGTAtcctgcacacgtcca
**1[151]**	GTACTATGGAACCCTAAACGGTAACCAAAAACtcctgcacacgtcca
**1[183]**	CCCGCCGCTGACGGGGCTGAGAGTTTAAATTGtcctgcacacgtcca
**1[215]**	TGTAGCGGGGAAGGGAGAGAGATCTCGCATTAtcctgcacacgtcca
**3[55]**	TGCTGGTAAACAGGAAGAATCCTGGTTTGCCCtcctgcacacgtcca
**3[87]**	GCCTGAGTTTTGACGCTAAAGGGAGGTGGTTCtcctgcacacgtcca
**3[119]**	CGTGCTTTCCTATAAACCCTTCGTTAGAACTCtcctgcacacgtcca
**3[151]**	CAGAGGTGTAATAAAATTATTTCAGTAATGTGtcctgcacacgtcca
**3[183]**	AATACCGAAGAACCCTATGACCCTGAAAGGCCtcctgcacacgtcca
**3[215]**	GAACTGATGTGGCACAATAAAGCTATGATATTtcctgcacacgtcca
**5[55]**	TTACAAACAACGTTATTCAATCAAAGGCCACCtcctgcacacgtcca
**5[87]**	AATACATTTATCATTTATCACCTTCAAATTAAtcctgcacacgtcca
**5[119]**	TGCAACAGTGCTGATTTTCTTCACGCTGATCAtcctgcacacgtcca
**5[151]**	CATCGGGACAGATGATTTTAGTTTTTGGGGCtcctgcacacgtcca
**5[183]**	GTCAGATGATTGTTTGACAGTTGATTCTACTAtcctgcacacgtcca
**5[215]**	TAAAGAAAGGTTAGAAAGTACGGTAAATCATAtcctgcacacgtcca

**7[55]**	GAATCCTTGAGAAGAGACAAACATACAGTTGAtcctgcacacgtcca
**7[87]**	TAAATCGTTAGGTCTGTTCAATTAAATATCTTtcctgcacacgtcca
**7[119]**	TTGCTTTGAATAGATTCTAATACCAAGTTCCAtcctgcacacgtcca
**7[151]**	TTAGTATCGCTGATGCAAATATCGCAGGATTAtcctgcacacgtcca
**7[183]**	CACCGGAACGCGAGAACAAAAAGATTGATAAGtcctgcacacgtcca
**7[215]**	TGTGATAAAATTTCATCTATTATAAGCTTAATtcctgcacacgtcca
**9[55]**	AAAATAATAATCAATAAGGCATTTTTTAACAAtcctgcacacgtcca
**9[87]**	TTTATCAACAAGAACGATTTAACATGGAAACAtcctgcacacgtcca
**9[119]**	CAGTATAAAGCGAGTGTATGTCAACGCTCAGAtcctgcacacgtcca
**9[151]**	TTACCAACTCGTAGGAAATAGCGACTGGATAGtcctgcacacgtcca
**9[183]**	TTTTGCACGCAAATCAACCCTCGTTAAATATTtcctgcacacgtcca
**9[215]**	TTTTGAAGCTAAGAACTACGAGGCAAACAGTTtcctgcacacgtcca
**11[55]**	AAGCCCAAGCTATCTTAAATGAAATAAAGTACtcctgcacacgtcca
**11[87]**	TATCAGAGAAGCAGATAAATAAGACCAGACGAtcctgcacacgtcca
**11[119]**	CTAATTTGCCAAGCTATGTTCGTTACAAATTAtcctgcacacgtcca
**11[151]**	GGGCGACACAAAAGAATGAATTACAAATCTACtcctgcacacgtcca
**11[183]**	ATTCATATTACGCAGTTTGAGATGCAACATTAtcctgcacacgtcca
**11[215]**	AATAAGTTTACATAAAGAGAAACATGAGATTTtcctgcacacgtcca
**13[55]**	GTCATAGCCAAAATCAAAAATCACTAACATAAtcctgcacacgtcca
**13[87]**	AGACTGTACCGCCTCCCGACTTGAAGAATTAAtcctgcacacgtcca
**13[119]**	GTAAATATTGACAGAGAAAGTCGGAAATTTTGtcctgcacacgtcca
**13[151]**	GGAGTGTACCACCAGAACGGTCAAAGACCAGGtcctgcacacgtcca
**13[183]**	AGTAAGCGCATTGACAGCTCCATGCAAGAGTAtcctgcacacgtcca
**13[215]**	TGGAAAGCTGGCCTTGGCCTGATATTACCCAAtcctgcacacgtcca
**15[55]**	CCGCCACCCAGAGCCAAGTATTAAAAGGCCGGtcctgcacacgtcca
**15[87]**	AGGTGTATAGCCCAATTATTTCGGTAGCAGCAtcctgcacacgtcca
**15[119]**	CAGTGCCTTGAATCAAACAGAGTAACAGTCCAtcctgcacacgtcca
**15[151]**	TTCACGTTGTAGCATTTTTGAGGACACTACGAtcctgcacacgtcca
**15[183]**	GAACAACTCGTAACGAATCGGAACGGCAAAAGtcctgcacacgtcca
**15[215]**	TCAACAGTACGTTAGTGGGATCGTACCCCCAGtcctgcacacgtcca

Lower cases represent the sequences which is complementary to the extended handles of aptamer Broccoli.

Supplemental Table S6. Sequences of two-layer rectangular DNA origami C24

**5[55]**	TTACAAACAACGTTATTCAATCAAAGGCCACCtcctgcacacgtcca
**5[87]**	AATACATTTATCATTTATCACCTTCAAATTAAtcctgcacacgtcca
**5[119]**	TGCAACAGTGCTGATTTTCTTCACGCTGATCAtcctgcacacgtcca
**5[151]**	CATCGGGACAGATGATTTTAGTTTTTTGGGGCtcctgcacacgtcca
**5[183]**	GTCAGATGATTGTTTGACAGTTGATTCTACTAtcctgcacacgtcca
**5[215]**	TAAAGAAAGGTTAGAAAGTACGGTAAATCATAtcctgcacacgtcca

**7[55]**	GAATCCTTGAGAAGAGACAAACATACAGTTGAtcctgcacacgtcca
**7[87]**	TAAATCGTTAGGTCTGTTCAATTAAATATCTTtcctgcacacgtcca
**7[119]**	TTGCTTTGAATAGATTCTAATACCAAGTTCCAtcctgcacacgtcca
**7[151]**	TTAGTATCGCTGATGCAAATATCGCAGGATTAtcctgcacacgtcca
**7[183]**	CACCGGAACGCGAGAACAAAAAGATTGATAAGtcctgcacacgtcca
**7[215]**	TGTGATAAAATTTCATCTATTATAAGCTTAATtcctgcacacgtcca
**9[55]**	AAAATAATAATCAATAAGGCATTTTTTAACAAtcctgcacacgtcca
**9[87]**	TTTATCAACAAGAACGATTTAACATGGAAACAtcctgcacacgtcca
**9[119]**	CAGTATAAAGCGAGTGTATGTCAACGCTCAGAtcctgcacacgtcca
**9[151]**	TTACCAACTCGTAGGAAATAGCGACTGGATAGtcctgcacacgtcca
**9[183]**	TTTTGCACGCAAATCAACCCTCGTTAAATATTtcctgcacacgtcca
**9[215]**	TTTTGAAGCTAAGAACTACGAGGCAAACAGTTtcctgcacacgtcca
**11[55]**	AAGCCCAAGCTATCTTAAATGAAATAAAGTACtcctgcacacgtcca
**11[87]**	TATCAGAGAAGCAGATAAATAAGACCAGACGAtcctgcacacgtcca
**11[119]**	CTAATTTGCCAAGCTATGTTCGTTACAAATTAtcctgcacacgtcca
**11[151]**	GGGCGACACAAAAGAATGAATTACAAATCTACtcctgcacacgtcca
**11[183]**	ATTCATATTACGCAGTTTGAGATGCAACATTAtcctgcacacgtcca
**11[215]**	AATAAGTTTACATAAAGAGAAACATGAGATTTtcctgcacacgtcca

Lower cases represent the sequences which is complementary to the extended handles of aptamer Broccoli.

Supplemental Table S7. Sequences of two-layer rectangular DNA origami C12 and C12adj

**Name**	**Sequences**
**5[87]**	AATACATTTATCATTTATCACCTTCAAATTAAtcctgcacacgtcca
**5[119]**	TGCAACAGTGCTGATTTTCTTCACGCTGATCAtcctgcacacgtcca
**5[151]**	CATCGGGACAGATGATTTTAGTTTTTGGGGCtcctgcacacgtcca
**5[183]**	GTCAGATGATTGTTTGACAGTTGATTCTACTAtcctgcacacgtcca
**7[87]**	TAAATCGTTAGGTCTGTTCAATTAAATATCTTtcctgcacacgtcca
**7[119]**	TTGCTTTGAATAGATTCTAATACCAAGTTCCAtcctgcacacgtcca
**7[151]**	TTAGTATCGCTGATGCAAATATCGCAGGATTAtcctgcacacgtcca
**7[183]**	CACCGGAACGCGAGAACAAAAAGATTGATAAGtcctgcacacgtcca
**9[87]**	TTTATCAACAAGAACGATTTAACATGGAAACAtcctgcacacgtcca
**9[119]**	CAGTATAAAGCGAGTGTATGTCAACGCTCAGAtcctgcacacgtcca
**9[151]**	TTACCAACTCGTAGGAAATAGCGACTGGATAGtcctgcacacgtcca
**9[183]**	TTTTGCACGCAAATCAACCCTCGTTAAATATTtcctgcacacgtcca

Lower cases represent the sequences which is complementary to the extended handles of aptamer Broccoli.

Supplemental Table S8. Sequences of two-layer rectangular DNA origami C12int

**Name**	**Sequences**
**1[55]**	CAAGAGTCGGGCGAAATGAGAGAGCCTCAGGAtcctgcacacgtcca
**1[119]**	TTGCGTATTGGCAAAGCCAGGGCGCCAGGGTAtcctgcacacgtcca

**1[183]**	CCCGCCGCTGACGGGGCTGAGAGTTTAAATTGtcctgcacacgtcca
**5[55]**	TTACAAACAACGTTATTCAATCAAAGGCCACCtcctgcacacgtcca
**5[119]**	TGCAACAGTGCTGATTTTCTTCACGCTGATCAtcctgcacacgtcca
**5[183]**	GTCAGATGATTGTTTGACAGTTGATTCTACTAtcctgcacacgtcca
**9[55]**	AAAATAATAATCAATAAGGCATTTTTAACAAtcctgcacacgtcca
**9[119]**	CAGTATAAAGCGAGTGTATGTCAACGCTCAGAtcctgcacacgtcca
**9[183]**	TTTTGCACGCAAATCAACCCTCGTTAAATATTtcctgcacacgtcca
**13[55]**	GTCATAGCCAAAATCAAAAATCACTAACATAAtcctgcacacgtcca
**13[119]**	GTAAATATTGACAGAGAAAGTCGGAAATTTTGtcctgcacacgtcca
**13[183]**	AGTAAGCGCATTGACAGCTCCATGCAAGAGTAtcctgcacacgtcca

Lower cases represent the sequences which is complementary to the extended handles of aptamer Broccoli.

Supplemental Table S9. Sequences of two-layer rectangular DNA origami C4

Name	Sequences
**7[119]**	TTGCTTTGAATAGATTCTAATACCAAGTTCCAtcctgcacacgtcca
**7[151]**	TTAGTATCGCTGATGCAAATATCGCAGGATTAtcctgcacacgtcca
**9[119]**	CAGTATAAAGCGAGTGTATGTCAACGCTCAGAtcctgcacacgtcca
**9[151]**	TTACCAACTCGTAGGAAATAGCGACTGGATAGtcctgcacacgtcca

Lower cases represent the sequences which is complementary to the extended handles of aptamer Broccoli.

Supplemental Table S10. Sequences of two-layer rectangular DNA origami C1, tailed-Broccoli and tailed-baby spinach

Name	Sequences
**7[119]**	TTGCTTTGAATAGATTCTAATACCAAGTTCCAtcctgcacacgtcca
**Tailed Broccoli**	GAGACGGUCGGGUCCAGAUAUUCGUAUCUGUCGAGUAGA GUGUGGGCUCUUUGGACGUGUGCAGG
**Tailed baby spinach**	GGUGAAGGACGGGUCCAGUAGUUCGCUACUGUUGAGUAGA GUGUGAGCUCCUUUUGGACGUGUGCAGG

Lower cases represent the sequences which is complementary to the extended handles of aptamer Broccoli.

Supplemental Table S11. The thermodynamic parameters (ΔH, ΔS, ΔG and Tm) of free Broccoli aptamers and Broccoli with hairpins, duplex and nanostructure handles.

Name	Sequence	ΔH (kcal/mol)	ΔS (cal/K*mol)	ΔG (kcal/mol)	Tm (ºC)
**Tailed Broccoli**	GAGACGGUCGGGUCCAGAUAUUCGUAUCUGUCGAGUAGAGUGUGGGCUCUUUUGGACGUGUGCAGG	-100.2	-309.44	-7.94	50.7

**S0+Broccoli**	AATGCGAAAGAGTAGGAATCATTTTtcctgcacacgtccaGAGACGGUCGGGUCCAGAUAUUCGUAUCUGUCGAGUAGAGUGUGGGCUCUUUUGGACGUGUGCAGG	-239.7	-721.46	-24.6	59.1
**M0+Broccoli**	GTCACAATTGGGGACTTCCGGTTTTtcctgcacacgtccaGAGACGGUCGGGUCCAGAUAUUCGUAUCUGUCGAGUAGAGUGUGGGCUCUUUUGGACGUGUGCAGG	-242.7	-729.84	-25.1	59.4
**H0+Broocli**	TTTtcctgcacacgtccaGAGACGGUCGGGUCCAGAUAUUCGUAUCUGUCGAGUAGAGUGUGGGCUCUUUUGGACGUGUGCAGG	-217.9	-648.54	-24.54	62.8
**H5+Broocli**	CGAGCTTTTGCTCGTTTtcctgcacacgtccaGAGACGGUCGGGUCCAGAUAUUCGUAUCUGUCGAGUAGAGUGUGGGCUCUUUUGGACGUGUGCAGG	-264.5	-783.49	-30.9	64.4
**H7+Broccoli**	CGAGCGATTTTTCGCTCGTTTtcctgcacacgtccaGAGACGGUCGGGUCCAGAUAUUCGUAUCUGUCGAGUAGAGUGUGGGCUCUUUUGGACGUGUGCAGG	-280.4	-825.89	-34.16	66.4
**H10+Broocli**	CGAGCGATCTTTTTAGATCGCTCGTTTtcctgcacacgtccaGAGACGGUCGGGUCCAGAUAUUCGUAUCUGUCGAGUAGAGUGUGGGCUCUUUUGGACGUGUGCAGG	-303.5	-890.61	-37.97	67.6
**H15+Broccoli**	CGTAGCAGAGCGACTTTTTAGTCGCTCTGCTACGTTTtcctgcacacgtccaGAGACGGUCGGGUCCAGAUAUUCGUAUCUGUCGAGUAGAGUGUGGGCUCUUUUGGACGUGUGCAGG	-346	-1005.14	-46.32	71.1
**1-1 handle +Broccoli**	TGAGTCTGGGGGCGGGTGCTTCAGACCAAGGTTCGCACCTTACTTTTtcctgcacacgtccaGAGACGGUCGGGUCCAGAUAUUCGUAUCUGUCGAGUAGAGUGUGGGCUCUUUUGGACGUGUGCAGG	-311.4	-933.46	-33.09	60.4
**C1 handle +Broccoli**	TTGCTTTGAATAGATTCTAATACCAAGTTCCAtcctgcacacgtccaGAGACGGUCGGGUCCAGAUAUUCGUAUCUGUCGAGUAGAGUGUGGGCUCUUU	-249.1	-747.91	-26.11	59.9

	UGGACGUGUGCAGG				

Lower cases represent the sequences which is complementary to the extended handles of aptamer Broccoli.
